# Ankle fractures: a systematic review of patient-reported outcome measures and their measurement properties

**DOI:** 10.1007/s11136-022-03166-3

**Published:** 2022-06-18

**Authors:** Michael Quan Nguyen, Ingvild Dalen, Marjolein Memelink Iversen, Knut Harboe, Aksel Paulsen

**Affiliations:** 1 Department of Orthopedic Surgery, Stavanger University Hospital, Helse Stavanger HF, Stavanger, Norway; 2 Department of Quality and Health Technology, Faculty of Health Sciences, University of Stavanger, Stavanger, Norway; 3 Department of Research, Stavanger University Hospital, Helse Stavanger HF, Stavanger, Norway; 4 Centre on Patient-Reported Outcomes, Department of Research and Development, Haukeland University Hospital, Helse Bergen HF, Bergen, Norway; 5 Department of Health and Caring Sciences, Faculty of Health and Social Sciences, Western Norway University of Applied Sciences, Bergen, Norway; 6 Department of Clinical Medicine, Faculty of Medicine, University of Bergen, Bergen, Norway; 7 Department of Public Health, Faculty of Health Sciences, University of Stavanger, Stavanger, Norway

**Keywords:** Patient reported outcome measures, Ankle fractures, Systematic review, Validity, Measurement properties

## Abstract

**Purpose:**

Ankle fractures are commonly occurring fractures, especially in the aging population, where they often present as fragility fractures. The disease burden and economic costs to the patient and society are considerable. Choosing accurate outcome measures for the evaluation of the management of ankle fractures in clinical trials facilitates better decision-making. This systematic review assesses the evidence for the measurement properties of patient-reported outcome measures (PROMs) used in the evaluation of adult patients with ankle fractures.

**Methods:**

Searches were performed in CINAHL, EMBASE, Medline and Google Scholar from the date of inception to July 2021. Studies that assessed the measurement properties of a PROM in an adult ankle fracture population were included. The included studies were assessed according to the COnsensus-based Standards for the selection of health Measurement INstruments (COSMIN) methodology for systematic reviews of PROMs.

**Results:**

In total, 13 different PROMs were identified in the 23 included articles. Only the Ankle Fracture Outcome of Rehabilitation Measure (A-FORM) presented some evidence on content validity. The Olerud-Molander Ankle Score (OMAS) and Self-reported Foot and Ankle Score (SEFAS) displayed good evidence of construct validity and internal consistency. The measurement properties of the OMAS, LEFS and SEFAS were most studied.

**Conclusion:**

The absence of validation studies covering all measurement properties of PROMs used in the adult ankle fracture population precludes the recommendation of a specific PROM to be used in the evaluation of this population. Further research should focus on validation of the content validity of the instruments used in patients with ankle fractures.

**Supplementary Information:**

The online version contains supplementary material available at 10.1007/s11136-022-03166-3.

## Introduction

Patients presenting with an ankle fracture is a common sight in the emergency department. A study demonstrated that approximately one of ten sustained fractures in patients older than 11 years are due to ankle fractures [1]. An epidemiological study on ankle fractures of the entire population in the United States estimated 673,214 cases over a period of five years, giving a incidence rate of 4.22/10,000 person years [2]. Ankle fractures occurs in all ages and both genders, but with a bimodal distribution curve, with the first peak in young men, and a second peak in older women [1]. The link between an increased risk of ankle fractures in the elderly population and a reduction in bone mineral density has been established [3], indicating that ankle fractures in the older female population are considered a predictor for fragility. With increases in life expectancy, it is likely that the frequency of fragility ankle fractures will also rise in the future [4]. Presumably, this will have implications for the management of ankle fractures, considering the challenging nature of fragility fractures and the increasing complexity of patients’ clinical status as they age [5, 6]. With such a heterogeneous patient population and enhanced focus on patient-specific treatment, treatment approaches also differs largely. The estimated cost of surgically treated ankle fractures per patient was $8688–20,414 (2016 USD), with a mean duration of unemployment of 53–90 days [7]. Alongside this trend within the field of orthopedic surgery, there is a need for more accurate outcome measures, reflected in the increased use of patient-reported outcome measures (PROMs) in clinical and research settings in the last decade [8, 9].

A patient-reported outcome (PRO) is defined as “any report of the status of a patient’s health condition that comes directly from the patient without interpretation of the patient’s response by a clinician or anyone else”, and PROMs are the instruments used to measure PROs [10]. The measurement properties of an instrument provide information on the validity, reliability and responsiveness of the instrument in the context of use, and content validity is considered the most important aspect [11]. A recent review identified the Olerud-Molander Ankle Score (OMAS) as the most commonly used primary outcome in the assessment of patients with ankle fractures in clinical trials [12]. The American Orthopedic Foot and Ankle Score (AOFAS), which is considered a partially patient-reported outcome measure, was the fourth most commonly used outcome score for ankle fracture patients. Other reviews found that the AOFAS was the most commonly used instrument in foot and ankle disorders [13, 14], regardless of repeated concerns with its measurement properties [15–17]. However, the quality of the instrument relies upon on the measurement properties and should be the main concern when choosing the outcome measure in research and for clinical use [9]. A recommendation on which PROM should be used in patients with ankle fracture based on current evidence on measurement properties is warranted.

This systematic review assesses the evidence for the measurement properties and the interpretability of PROMs used in the evaluation of adult patients with ankle fractures and adheres to the COnsensus-based Standards for the selection of health Measurement INstruments (COSMIN) guidelines [11, 18, 19]. It takes into consideration the limitations from previously published systematic reviews [20, 21] by including validation studies of all PROMs and studies in a population mainly composed of ankle fracture patients. This will ensure an adequate representation of the target population and provide a more complete overview of the PROMs validated for use in this context.

## Methods

### Protocol and registration

The reporting of this review followed the checklist provided in the Preferred Reporting Items for Systematic reviews and Meta-Analyses (PRISMA) statement [22, 23]. The protocol has been submitted to the International Prospective Register of Systematic Reviews (PROSPERO) (registration number: CRD42019122800).

### Eligibility criteria

Studies that assessed the measurement properties of PROMs in an adult ankle fracture population with the Arbeitsgemeinschaft für Osteosynthesefragen/Orthopaedic Trauma Association (AO/OTA) classification 44 [24], including medial malleolar fracture, were selected for the current systematic literature review. The included studies involved a study population of at least 50% patients with ankle fractures.

The exclusion criteria were as follows: (1) articles in languages other than English or a Scandinavian language; (2) validation of a PROM against a non-PROM instrument, as these studies provide only indirect information on the measurement properties; and (3) proxy-reported PROMs, as these were considered observer-reported outcomes [10].

### Data sources and search strategy

A literature search was performed in Medline, EMBASE and CINAHL from the inception of the databases to the 6^th^ of July 2021. Three filters were applied: (1) a PROM-inclusion filter developed by the University of Oxford [25], (2) a validated sensitive search filter for measurement properties by Terwee et al. [26] and (3) an age filter to exclude results indexed with child and adolescent age groups only. A separate search in Google Scholar was performed with the following search phrase: *“ankle fracture” validation “patient reported outcomes” “measurement properties”*. The search strategy was devised in collaboration with expert research librarians and details are presented in Online Resource 1.

### Selection process

The review team consisted of four reviewers. The results from the search strategy were uploaded to Covidence [27]. All titles and abstracts were randomly screened for potential eligibility by two reviewers independently. Any disagreements were discussed between the two reviewers, and if in doubt, the full text was retrieved. The full text was retrieved for all abstracts that were potentially eligible for inclusion and again independently screened by two reviewers. Any disagreements were discussed between the two reviewers, and if consensus was not achieved, a third reviewer was consulted.

The initial screening included screening PROMs used in a more general fracture population. Two reviewers separately performed a secondary final screening of the included articles to retrieve studies limited to those meeting the eligibility criteria for the ankle fracture review.

The first author screened the references of the included articles for potential eligible studies.

### Data extraction

The extracted outcome variables were (1) content validity, including PROM development; (2) structural validity; (3) internal consistency; (4) cross-cultural validity/measurement invariance; (5) reliability; (6) measurement error; (7) criterion validity; (8) hypothesis testing for construct validity; (9) responsiveness; and (10) interpretability.

### Assessing the methodological quality of the studies

The COSMIN Risk of Bias checklist [19] was applied for the assessment of the methodological quality of the studies. The list contained questions for each measurement property, and each question was given a rating of very good, adequate, doubtful or inadequate. The overall rating for each measurement property per study followed the “the worse score counts” principle.

### Ratings of PROM development and content validity

PROM development was not considered a measurement property but was taken into account in the assessment of content validity and consisted of (1) PROM design, which accommodates concept elicitation and item generation, and (2) testing of the new PROM, which refers to a cognitive interview or a pilot study. It was a prerequisite when rating the PROM development that the methodological quality did not have an inadequate rating when rating the results against the criteria for good measurement properties.

The evaluation of content validity included three aspects: (1) relevance, (2) comprehensibility, and (3) comprehensiveness. For translations, only the comprehensibility aspect was assessed. Each aspect was rated sufficient, insufficient or indeterminate. PROMs that included the target population for the current review in the development phase were also given a content validity rating by the reviewers.

The results from the development study, content validity studies and reviewers’ ratings were summarized, and an overall rating of sufficient, insufficient or inconsistent was obtained based on the criteria for good content validity [11].

### Rating of the remaining measurement properties

The remaining measurement properties were assessed according to the COSMIN criteria for good measurement properties [18], resulting in a rating of sufficient, insufficient or indeterminate per study. Subsequently, the results from all studies on each measurement property were summarized and again rated against the COSMIN criteria for good measurement properties to yield an overall rating of sufficient, insufficient, inconsistent or indeterminate. In the assessment of the methodological quality of studies, twenty percent of the included articles were randomly selected to be independently assessed by two reviewers. Any disagreements or difficulties in ratings were discussed to achieve consensus. If this was not reached, a third reviewer was consulted.

The review team agreed that there are no gold standards in the evaluation of construct validity, except when comparing a shortened version against its original version [28]. Rather, hypotheses were formulated for the validation of construct validity. As there were no limitations to which PROMs were included in this review, it was not feasible to define hypotheses for every possible scenario a priori. Instead, threshold categories for correlations and a ground set of hypotheses were constructed (Online resource 2) [29]: instruments measuring (1) the same construct were expected to have at least moderate to high correlation (r > 0.6), (2) moderate correlation for related constructs (0.3 < r < 0.7), and (3) weak to moderate correlation for weakly related constructs (0.2 < r < 0.4). More specific hypotheses were formulated throughout the review with the expected direction and magnitude of the correlation depending on the construct of each instrument (Online Resource 3).

A similar approach was used in the assessment of responsiveness, but hypotheses were formed based on the expected correlation between the change scores of the instruments. The threshold categories for correlation was expected to be lower for change scores when compared to the scores of instruments at a single time point [30]. When the comparator instrument measure the same construct as the instrument under study, the correlation was expected to be high (r ≥ 0.5). If the comparator instrument measure a related construct, the correlation was expected to be moderate (0.3 < r < 0.5). For external measures with a dichotomous variable, an area under the curve (AUC) of 0.7 or above indicated sufficient ability of the instrument to discriminate between patients who improved and patients who did not improve according to the external measure of change.

### Interpretability

Interpretability is not considered a measurement property but refers to “the degree one can assign qualitative meaning” to the PROM score or change in PROM score [31], and is used as additional information when choosing the instrument. Data on distribution of scores, rate of missing items, floor/ceiling effect and minimal important change (MIC) were extracted.

### Quality of evidence

The modified Grading of Recommendations, Assessment, Development and Evaluation (GRADE) approach [18, 32] was applied to the summarized results to yield a grading for the quality of evidence. This grading expresses the level of certainty for the summarized results. Each measurement property received a grading of high, moderate, low or very low depending on four factors: (1) risk of bias; (2) inconsistency in the results across studies; (3) imprecision, which referred to the total sample size; and (4) indirectness, i.e., if evidence was derived from different populations or from the context of use.

### Recommendations

PROMs in category A are recommended for use in the evaluation of patients with ankle fractures. These PROMs exhibit evidence for sufficient content validity and at least low quality evidence for internal consistency. If there is good evidence for an insufficient measurement property, the PROM is disapproved for use and placed in category C. The remaining PROMs are placed in category B. These could be recommended by obtaining more evidence on sufficient measurement properties with further validation [18].

## Results

### Study selection

**Fig. 1:**
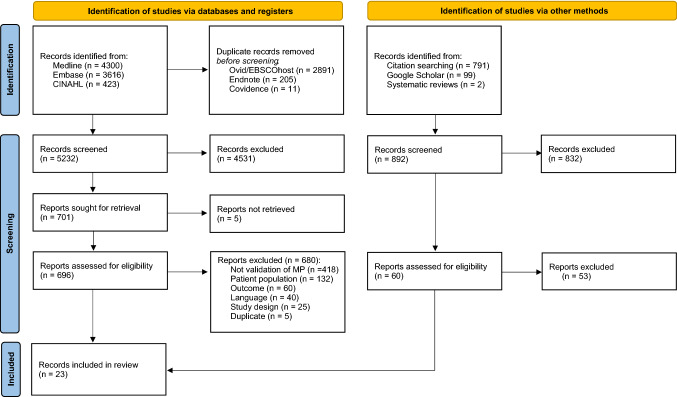
PRISMA flow diagram for the search strategy and selection of records. Template from: Page MJ, McKenzie JE, Bossuyt PM, Boutron I, Hoffmann TC, Mulrow CD, et al. The PRISMA 2020 statement: an updated guideline for reporting systematic reviews. BMJ 2021;372:n71. 10.1136/bmj.n71

Of the 8339 potential articles for this review, 3107 duplicates were identified and removed before screening commenced. The titles and abstracts of the remaining 5232 articles were screened for eligibility, and 4531 articles were excluded. In the next step, 696 full-estext articles were screened by the inclusion/exclusion criteria, and 680 articles were excluded. Five articles were included from the screening of references in the included articles [33–37], one article was included based on a Google Scholar search [38], and one article [39] was included based on a systematic review [21]. In total, 23 articles were included in the review (Fig. 1).

### Study characteristics

Thirteen PROMs were identified (Table 1) and characteristics of PROMs under study are reported in Table 2. Comparator instruments identified in the studies are described in online resource 4. In the 23 articles included, 28 studies were described. For some of the articles, multiple studies were described assessing different measurement properties in the same article. Eleven studies included only surgically treated ankle fractures. Patient ages ranged from 16–94, with a mean age 41–58. Follow-up times ranged from one month to five years (Table 3).

**Table 1:** Included PROMs

Category	PROM
Condition-specific	American Academy of Orthopaedic Surgeons Foot and Ankle Outcomes Questionnaire (AAOS-FAOQ) Ankle Fracture Outcome of Rehabilitation Measure (A-FORM) Foot and Ankle Ability Measure (FAAM) Lower Extremity Functional Scale (LEFS) Munich Ankle Questionnaire (MAQ) Olerud-Molander Ankle Score (OMAS) Self-reported Foot and Ankle Score (SEFAS) Visual Analogue Scale Foot and Ankle (VAS-FA)
Generic	Short Musculoskeletal Function Assessment (SMFA) Trauma Expectation Factor Trauma Outcome Measure (TEFTOM) Western Ontario and McMaster Universities Osteoarthritis Index (WOMAC), version 3.0, foot and ankle
Patient-Reported Outcomes Measurement Information System Computer Adaptive Test (PROMIS CAT)	PROMIS-Lower Extremity (LE) CAT PROMIS-Physical Function (PF) CAT, version 1.2

**Table 2:** Characteristics of included PROMs

PROM	Reference	Construct	Target population	(Sub)scale(s) / number of items	Response options	Range of scores/scoring	Original language	Available translations^a^	Cost
American Academy of Orthopaedic Surgeons foot and ankle outcomes questionnaire, (1) the global foot and ankle scale, (2) the shoe comfort scale	Zelle 2017	(1) Symptoms and functional status related to the foot and ankle (2) Ability to wear a variety of shoe types comfortably	Foot and ankle problems	The global foot and ankle scale / 20 items. The shoe comfort scale / 5 items	The global foot and ankle scale: 1–3, 1–5, 1–6, 1–7. The shoe comfort scale: Yes/no	0–100% (best)	English	Spanish	Free, register at AAOS for scoring algorithm
Ankle Fracture Outcome of Rehabilitation Measure v1.0	McPhail 2014	Life impacts (physical, social and psychological recovery) after ankle fracture	Ankle fracture	15 items (summary only for 14 items)	1–5	1–100	English	?	Contact developer
Foot and Ankle Ability Measure	Schultz 2020	Physical function (disability)	Musculoskeletal disorders of the feet and ankle	ADL + sports / 21 + 8 items	0–4	ADL 0–84 (worst), sports 0–32 (worst)	English	Chinese, Danish, Dutch, German, Japanese, Persian, Spanish, Thai, Turkish	Free
Lower Extremity Functional Scale	Garratt 2018, Lin 2009, Repo 2017, Repo 2019	Physical function (disability)	Musculoskeletal conditions or disorders in lower limb	20 items	0–4	0–80 (best)	English	Arabic, Brazilian Portuguese, Canadian, Chinese, Italian, Dutch, Finnish, French, Malaysian, Persian, Spanish, Turkish	Free
Munich Ankle Questionnaire	Greve 2018	Recovery/follow-up of ankle pathology	Ankle disorders	3 subscales (pain, daily living/work and movement/ROM) / 12 items	Pain 1–10; Daily living 1–10; Work 1–7; Movement 1–3; ROM 0–20	106 points (best), 0–100% (best)	German	?	?
Olerud-Molander Ankle Score	Büker 2017, Nilsson 2013, Garratt 2018, McKeown 2021, Ponzer 1999, Olerud Molander 1984, Turhan 2017, Shah 2007, Lash 2002	Physical disability	Follow-up of ankle fracture	1 scale / 9 items	Pain (0–25); Stiffness (0–10); Swelling (0–10); Stair climbing (0–10); Running (0–5); Jumping (0–5); Use of supports (0–10); Work/activity (0–20)	0–100 (best)	English	Norwegian, Swedish, Turkish	Free, contact developer
PROMIS LE CAT	Gausden 2018	Physical function in patients with lower extremity conditions	Patients with lower extremity conditions	79 items in question bank	0–4	Standardized score, mean 50 SD 10	English	Danish, Dutch, Finnish, Portuguese (Brazil)	Depends on software
PROMIS PF ver 1.2 CAT	Gausden 2018	Physical function	General	124 items in question bank	0–4	Standardized score, mean 50 SD 10	English	Danish, Dutch, Finnish, Portuguese (Brazil)	Depends on software
Self-Reported Foot and Ankle Score	Garratt 2018, Erichsen 2021	Pain, function, other	Osteoarthritis and inflammatory arthritis of the ankle and outcome of ankle surgery	12 items	0–4	Garratt: 12–60 (worst); Erichsen 0–48 (best)	Swedish	Danish, English, French, German, Spanish, Turkish	?
Short Musculoskeletal Functional Assessment	Obremskey 2007	Physical function (dysfunction index) and impact of limitation of function (bother index)	Musculoskeletal conditions	2 subscales: Dysfunction index (34 items) Bother index (12 items)	1–5	Converted to 0–100 (worst)	English	Chinese, Dutch, French, German, Korean, Japanese, Portuguese, Spanish, Swedish	Free
Trauma Expectation Factor Trauma Outcomes Measure	Suk 2013, Fang 2020	Pain, physical function, disability, injury satisfaction and overall satisfaction. 2 parts: TEF—expectations, TOM—outcome	General orthopedic trauma patients	2 parts × 10 items per part, 5 domains, 1 scale	0–4	0–40 (best)	English	Portuguese	?
Visual Analogue Scale Foot and Ankle	Repo 2018	Not defined	Foot and ankle patients	Three subscales (function, pain, other complaints) / 20 items	VAS 0–100 mm	0–100 (best)	German	English, Thai, Indian (Malayalam), Finnish	Free
Western Ontario and McMaster Universities Osteoarthritis Index ver. 3.0 ankle/foot	Ponkilainen 2019	Physical disability and symptoms	Osteoarthritis in foot/ankle	Three subscales (pain, stiffness and physical function) / 24 items	VAS 0–100	Index 0–100 (worst)	English	91	License required

^a^Not exhaustive list

*PROMIS CAT* Patient-Reported Outcomes Measurement Information System Computer Adaptive Test, *PF* Physical function, *LE* Lower extremity, *ADL* Activities of daily living, *?* Unknown

**Table 3:** Characteristics of the included studies

PROM	References	N	Age, mean ± SD (range)	Sex, % female	Patient selection	Ankle Fx (%)	Surgical Tx (%)	Follow-up	Country	PROM Language	Response rate
AAOS-FAOQ	Zelle 2017 [39]	83; test–retest: 63	?	?	Ankle or foot disorder	> 49	?	?	Mexico	Spanish	83% (83/100)
A-FORM v1.0	McPhail 2014 [41]	41	Median 37 (IQR: 28)	27	Ankle Fx	90	46	12–16 weeks	?	English	80% (41/51)
FAAM	Schultz 2020 [51]	27		57	Ankle Fx, pilon, distal tibia	78	100	42 weeks ± 4.2	USA	English	?
LEFS	Lin 2009 (1) [44]	306	45 ± 16	50	Ankle Fx	100	57	26 weeks	Australia	English	?
LEFS	Lin 2009 (2) [44]	60	49 ± 17	52	Ankle Fx	100	?	26 weeks	Australia	English	?
LEFS	Lin 2009 (3) [44]	Baseline to 4 weeks: 233; Baseline to 24 weeks: 90	?	?	Ankle Fx	100	?	4 and 24 weeks	Australia	English	?
LEFS	Repo 2017 [34]	166	55 ± 16	53	Foot or ankle surgery	> 73	100	Mean: 4 years (range: 0–14)	Finland	Finnish	22% (166/747)
LEFS	Repo 2019 [37]	182	55 ± 16	54	Foot or ankle surgery	73	100	3.2 years ± 9.6	Finland	Finnish	?
MAQ	Greve 2018 [52]	148	Median 45 ± 16	53	Ankle disorders	51	?	4 months	Germany	German	Test–retest: 73% (118/162); construct validity: 88% (142/162); responsiveness: 57% (92/162)
OMAS	Büker 2017 [53]	91	42 ± 13 (20–60)	28	Ankle Fx	100	100	28 ± 8.9 months	Turkey	Turkish	?
OMAS	Lash 2002 [54]	74	49	70	Ankle Fx	100	69	2 years	New Zealand	English	52% (74/141)
OMAS	McKeown 2021 [45]	620	46 ± 17 (18–94)	56	Ankle Fx	100	53	3.5 months	UK	English	?
OMAS	Nilsson 2013 (1) [50]	42	42 ± 14	55	Ankle Fx,	100	100	12 months	Sweden	Swedish	?
OMAS	Nilsson 2013 (2) [50]	6 months: 105; 12 months: 99	44 ± 14	60	Ankle Fx,	100	100	12 months	Sweden	Swedish	?
OMAS	Nilsson 2013 (3) [50]	46	43 ± 14	56	Ankle Fx,	100	100	12 months	Sweden	Swedish	?
OMAS	Olerud-Molander 1984 [33]	90	?	?	Ankle Fx, multicomponent Fxs,	100	100	?	?	?	?
OMAS	Ponzer 1999 [55]	41	41 ± 12	54	Ankle Fx	100	100	27	Sweden	?	77% (41/53)
OMAS	Shah 2007 [38]	69	51 (20–81)	62	Ankle Fx	100	100	5 years	UK	English	81% (69/85)
OMAS	Turhan 2017 [36]	100	42 ± 18 (16–81)	49	Ankle Fx	100	57	4.3 years	Turkey	Turkish	?
OMAS, LEFS, SEFAS	Garratt 2018 [40]	567; test–retest: 182	58 (22–91)	57	Ankle Fx	100	100	4.3 years, IQR: 3.9–5.1	Norway	Norwegian	59% (567/959); test–retest: 60% (182/299)
PROMIS PF v1.2/LE CAT	Gausden 2018 [56]	132	47 ± 18	60	Ankle Fx	100	100	12	USA	English	85% (132/156)
SEFAS	Erichsen 2021 (1) [43]	10	?	?	Ankle disorder	?	?	?	Denmark	Danish	
SEFAS	Erichsen 2021 (2) [43]	125	49 (18–81)	55	Ankle-related Fxs	68	79	6 weeks	Denmark	Danish	87% (125/143)
SEFAS	Erichsen 2021 (3) [43]	85	53 (19–81)	59	Ankle-related Fxs	100	69	7.2 weeks	Denmark	Danish	59% (85/143)
SMFA	Obremskey 2007 [57]	127	48 ± 18 (17–85)	58	Ankle Fx,	100	100	Mean: 27 ± 17 months (range: 6–64 months)	?	English	?
TEFTOM	Fang 2020 [58]	193	44 (17–81)	47	Ankle Fx	90	100	12 months	Spain, Germany, Switzerland, China, India	German, Spanish, Chinese	?
TEFTOM	Suk 2013 [42]	148	41 ± 15	45	Isolated ankle or distal tibia Fx	90	100	20 months	Brazil, Canada, USA	Portuguese, English	74% (148/201)
VAS-FA	Repo 2018 [35]	165	56 ± 16	55	Foot or ankle surgery	83	100	Mean: 4.3 years ± 4.7	Finland	Finnish	?
WOMAC	Ponkilainen 2019 [59, 60]	130	56 ± 17	57	Ankle Fx	100	100	Mean: 4 years (range: 1 month-14 years)	Finland	Finnish	61% (130/212)

*Fx* fracture, *Tx* treatment, *?* not reported, *IQR* interquartile range

### Measurement properties

One article assessed the measurement properties of several PROMs [40]. Most of the studies assessed multiple measurement properties. No studies assessed cross-cultural validity/measurement invariance or criterion validity. The measurement properties of the OMAS were most frequently assessed. Table 4 presents the results from the Ankle Fracture Outcome of Rehabilitation Measure (A-FORM) and the three most validated instruments. A summary of findings table for all PROMs is presented in Online Resource 5.

**Table 4:** Summary of findings tables for the A-FORM, LEFS, OMAS and SEFAS

					PROM: A-FORM									
References	Content validity		Structural validity		Internal consistency		Reliability		Measurement error		H testing for construct validity		Responsiveness	
	MQ	R	MQ	R	MQ	R	MQ	R	MQ	R	MQ	R	MQ	R
McPhail 2014 [40]	I	+												

Summarized results	NA													
Overall rating	Sufficient													
Quality of evidence	Very low; PROM development study inadequate, only reviewers’ ratings													

					PROM: LEFS									
References	Content validity		Structural validity		Internal consistency		Reliability		Measurement error		H testing for construct validity		Responsiveness	
	MQ	R	MQ	R	MQ	R	MQ	R	MQ	R	MQ	R	MQ	R
Garratt 2018 [49]			V	( +) CFA: CFI 0.99/TLI 0.99/RMSEA 0.091	D	( +) Cα = 0.96	A	( +) ICC: 0.91	A	(?) SDC: 12.5	A	H met: 7 H unmet: 1		
Garratt 2018 [49]											D	H unmet: 1		
Lin 2009 (1) [42]			V	(-)	D	( +) Cα = 0.90–0.96								
Lin 2009 (2) [42]											V	H met: 2		
Lin 2009 (3) [42]													D	H met: 2 AUC: 0.79 and 0.84
Repo 2017 [34]	D	+	A	?	D	Cα = 0.96	A	( +) ICC: 0.93	A	?	A	H met: 4		
Repo 2019 [37]			A	-										

Summarized results	Only comprehensibility		±		Cα: 0.90–0.96		ICC: 0.91–0.93		SDC: 12.5 MIC: unknown		H met: 13 H unmet: 2		H met: 2	
Overall rating	NA		Inconsistent		Sufficient		Sufficient		Indeterminate		Sufficient 87% confirmed		Sufficient	
Quality of evidence	NA		NA		NA; due to lack of evidence for sufficient structural validity		High; two studies of adequate quality		NA		High		Low	

					PROM: OMAS									
Reference	Content validity		Structural validity		Internal consistency		Reliability		Measurement error		H testing for construct validity		Responsiveness	
	MQ	R	MQ	R	MQ	R	MQ	R	MQ	R	MQ	R	MQ	R
Büker 2017 [44]					V	( +) Cα = 0.76	I	( +) ICC: 0.94			A	H met: 6		
Lash 2002 [45]											I	NA		
Garratt 2018 [49]			V	( +) CFA: CFI 0.99/TLI 0.98/RMSEA 0.087	V	( +) Cα = 0.82	A	( +) ICC: 0.92	A	(−) SDC: 19.0; MIC: 9.7^a^	A	H met: 6; H unmet: 2		
Garratt 2018 [49]											D	H unmet: 1		
McKeown 2021 [46]			A	(?)	V	( +) Cα total: 0.76					A	H met: 8; H unmet: 4		
Nilsson 2013 (1) [47]					V	( +) Cα = 0.76	D	( +) ICC: 0.94	D	(−) SDC: 12.0; MIC: 9.7^a^				
Nilsson 2013 (2) [47]											I	NA		
Nilsson 2013 (3) [47]											A	H met: 5		
Olerud-Molander 1984 [33]											I	NA		
Ponzer 1999 [48]											D	H met: 8; H unmet: 2		
Shah 2007 [38]											A	H met: 1; H unmet: 1		
Turhan 2017 [36]	D	+			V	( +) Cα = 0.84	I	( +) ICC: 0.98	I	( +) SDC: 9.1; MIC: 9.7^a^	A	H met: 2; H unmet: 2		

Summarized results	Only comprehensibility		Unidimensional		Cα: 0.76–0.84		ICC: 0.92–0.98		SDC: 9.1–19.0; MIC: 9.7^a^		H met: 36; H unmet: 12			
Overall rating	NA		Sufficient		Sufficient		Sufficient		Insufficient		Sufficient 75% confirmed			
Quality of evidence	NA		High; one very good study		High; multiple very good studies; consistent results		Moderate; only one study of adequate quality		Very low; only one study of adequate quality, one MIC, and indirectness (follow-up time 4.3 years vs. 16 weeks)		High; multiple studies of adequate quality			

					PROM: SEFAS									
References	Content validity		Structural validity		Internal consistency		Reliability		Measurement error		H testing for construct validity		Responsiveness	
	MQ	R	MQ	R	MQ	R	MQ	R	MQ	R	MQ	R	MQ	R
Erichsen 2021 (1) [51]	I	+												
Erichsen 2021 (2) [51]					V	( +) Cα = 0.93					D	H unmet: 1		
Erichsen 2021 (3) [51]							I	( +) ICC: 0.93	I	(−) SDC: 6.8; MIC: 5				
Garratt 2018 [49]			V	( +) CFA:CFI 0.99/TLI 0.99/RMSEA 0.063	V	( +) Cα = 0.93	A	( +) ICC: 0.93	A	(−) SDC: 6.6; MIC: 5^b^	A	H met: 6; H unmet: 2		

Summarized results	Only comprehensibility rated		Unidimensional		Cα = 0.93		ICC: 0.93		SDC: 6.6–6.8; MIC: 5^b^		H met: 6; H unmet: 3			
Overall rating	NA		Sufficient		Sufficient		Sufficient		Insufficient		Sufficient 75% confirmed (based on at least adequate MQ: H met: 6, H unmet: 2)			
Quality of evidence	NA		High		High		Moderate; only one study of adequate quality		Very low; MIC based on only one study and not adequately performed		Moderate			

No data on the validation of cross-cultural validity/measurement invariance or criterion validity in the studies. These measurement properties have been removed from the table.

^a^MIC from study of McKeown et al.

^b^MIC from study of Erichsen et al.

*H* Hypothesis, *MQ* methodological quality, *R* rating, *V* very good, *A* adequate, *D* doubtful, *I* inadequate, *+* sufficient, *?* indeterminate, − insufficient, *NA* not applicable, *CFI* comparative fit index, *TLI* Tucker–Lewis index, *RMSEA* root mean square error of approximation, *Cα* Cronbach’s alpha, *ICC* intraclass correlation coefficient, *SDC* smallest detectable change, *MIC* minimal important change, *AUC* area under curve

### PROM development and content validity

Only the A-FORM [41] had a methodologically adequate PROM design, but a lack of cognitive interviews or pilot studies yielded an inadequate rating for methodological quality regarding the total PROM development. The Trauma Expectation Factor Trauma Outcome Measure (TEFTOM) [42] was rated as having inadequate methodology in both PROM design and pilot study measures. Due to inadequate ratings regarding total PROM development, the ratings of both instruments were based on reviewers’ ratings only and achieved the lowest level of evidence.

Three studies included translations [35, 36, 43] and were assessed for comprehensibility as part of the content validity study but were not given a total content validity rating or quality of evidence grading due to lack of validation on the relevance and comprehensiveness aspects.

### Structural validity

One study performed confirmatory factor analysis (CFA) on the OMAS, Self-reported Foot and Ankle Score (SEFAS) and Lower Extremity Functional Scale (LEFS) [40], and each met the criteria for a sufficient rating of structural validity (Table 4). However, two other studies demonstrated lack of unidimensionality for the LEFS with Rasch analysis [37, 44]. In addition, exploratory factor analysis (EFA) was performed to explore the dimensionality of the OMAS [45] and LEFS [34], and two subscales were identified in both instruments. The summarized results for the LEFS are conflicting, and the level of evidence was not graded. As the COSMIN guidelines do not define criteria for EFA, the result of these studies did not receive a rating.

### Internal consistency

Summarized results from several studies of very good methodological quality yielded a Cronbach’s alpha of 0.76–0.84 and 0.93 for the OMAS and SEFAS, respectively, indicating sufficient internal consistency. Internal consistency parameters were reported for the LEFS and Western Ontario and McMaster Universities Osteoarthritis Index (WOMAC), but these were not rated due to a lack of evidence for sufficient structural validity.

### Reliability

The LEFS achieved high quality evidence for sufficient reliability, supported by two studies with adequate methodological quality reporting intraclass correlation coefficient (ICC) of 0.91–0.93 (Table 4). The OMAS, SEFAS and Visual Analogue Scale Foot and Ankle (VAS-FA) had moderate evidence for sufficient reliability, while the TEFTOM and Munich Ankle Questionnaire (MAQ) had low and very low evidence for sufficient reliability, respectively.

### Measurement error

The summarized result of the smallest detectable change (SDC) for the OMAS was 9.1–19.0, One study of inadequate methodological quality reported a value of 9.1 and was less decisive for the overall rating. The remaining studies reported values of 12.0 and 19.0, which was higher than the MIC of 9.7 points reported by McKeown et al. This indicated that the instrument cannot separate an important change (from the patients’ perspective) from measurement error between these values, resulting in an insufficient rating. The quality of evidence was downgraded to very low for three reasons: (1) presence of only one study assessing the MIC, (2) only one study of adequate methodological quality, and (3) indirectness due to considerable differences in follow-up times (16 weeks and 4.3 years) (Table 4).

Two studies on the SEFAS provided SDCs between 6.6 and 6.8 [40, 43], with one exhibiting inadequate methodological quality due to lack of stability between measurement points. Both studies reported SDCs to be higher than the MIC based on five points reported in the study by Erichsen et al. [43], but the calculation of this MIC carries a considerable risk of bias due to low sample size and inconsistency in the change score across subgroups.

The LEFS lacked MIC reporting and could not be rated according to the criteria for good measurement properties.

### Hypothesis testing for construct validity

The OMAS, WOMAC, LEFS, SEFAS and MAQ had 75% or more confirmed hypotheses. Validation of the Finnish version of the VAS-FA was not a clearly defined construct, and the study was rated as having inadequate methodological quality. The TEFTOM and PROMIS-PF CAT were the only instruments with an insufficient overall rating, however, the quality of evidence was low.

### Responsiveness

The LEFS achieved a sufficient rating with two confirmed hypotheses (Table 4). The authors used an external measure but did not specify the question that resulted in a downgrading of the level of evidence. Regarding the MAQ, three hypotheses were confirmed based on the construct approach, correlating the three domains to the same GRS and yielding a sufficient rating with moderate quality of evidence.

### Interpretability

The MICs of the OMAS and SEFAS were 9.7 and five points, respectively. The latter included a small sample size of 39 patients, and the data did not present a gradual increase in change scores among patients who improved, which introduces risk of bias in the determination of this value.

A floor effect of 22.4% was reported with the SEFAS at the six-week follow-up. A ceiling effect was reported for the OMAS (17%) and LEFS (27–29%), where both studies had a follow-up time of more than four years (Online Resource 6).

## Discussion

### Summary of evidence

A recent review of PROMs used as primary outcomes in interventional trials for patients with ankle fractures [12] identified the OMAS as the most commonly used multi-item PROM. In a systematic review assessing measurement properties of PROMs used in foot and ankle disease, the Manchester-Oxford Foot Questionnaire (MOXFQ) was reported to have the best overall psychometric properties [46]. However, the current review illustrates that the MOXFQ is completely absent in validation studies for the ankle fracture population. Collectively, there is still a lack of studies covering all measurement properties of PROMs for patients with ankle fractures. Among the PROMs used in the evaluation of the ankle fracture population, the measurement properties and interpretation of the OMAS, LEFS and SEFAS were most studied. However, there is a consistent lack of validation of the most important measurement property, i.e., content validity, reflecting the uncertainty in covering all aspects of a given construct. Thus, none of the PROMs could be categorized in category A.

### Validity and reliability

The OMAS was most frequently assessed PROM in this study population but was missing a content validity study of good quality. Despite inadequate methodology in PROM development, subsequent content validity studies could provide evidence for sufficient content validity. Of the instruments included in this review, only the A-FORM had an adequate PROM design [41]. The design and concept elicitation were based on a qualitative study on the life impact of ankle fractures [47] but lacked cognitive interviews or pilot tests. The developers of this instrument complied with many of the crucial steps in the development phase of a PROM, rendering a sound foundation for subsequent validation studies. The TEFTOM [42], on the other hand, had severe flaws in the development phase, where the study population was limited to fractures of the ankle and distal tibia. Such a limitation will not suffice to provide an adequate representation of the instrument’s intended population of general trauma patients.

In regard to structural validity, CFA is preferred over EFA for testing existing factor structures [48]. The OMAS and SEFAS appeared to be unidimensional when assessed with CFA [40]. However, the OMAS was also assessed with EFA [45], and two subscales were found, namely, (1) ankle function and (2) ankle symptoms, which indicates a bifactor structure in this instrument.

The LEFS was also assessed with CFA and achieved a sufficient rating of structural validity, but data from the same study showed a better fit with a bidimensional structure [40]. Another study validating the Finnish version of the instrument also found a two-factor structure [34]. Lin et al. [44] performed a Rasch analysis of the LEFS at three different time points. Most of the items were within the acceptable range for goodness-of-fit, but one item (sitting for 1 h) had unacceptable outfit statistics at all time points. The article did not provide enough information for a rating based on criteria for good measurement properties [49], but the Rasch analysis showed a lack of items for patients with greater abilities, drawing attention toward the cautious use of the instrument in patients with high demands or long-term follow-up of ankle fractures. Another Rasch analysis of the LEFS demonstrated disordered item thresholds for the response categories [37]. These studies had a methodological quality of at least adequate rating, but the results were conflicting. No obvious separation of studies into subgroups was identified that could explain the discrepancies. If this instrument was to be used in an ankle fracture population, one should be wary of the possible lack of unidimensionality.

Reliability and measurement error are usually assessed with a test–retest study. Often, the measurement error of an instrument is neglected, and reliability is tested only by providing an ICC. However, the assessment of measurement error, together with MIC values, permits another dimension to the interpretation of the statistical and clinical meaning of the scores. In the current review, the OMAS, SEFAS and LEFS displayed good evidence of sufficient reliability. Measurement error parameters for these instruments were reported, but the lack of MIC values for these instruments in the ankle fracture population made the interpretation incomplete. As an example, only one study reported the MIC for the OMAS [45]. When evaluated together with two other studies that reported an SDC larger than the MIC [40, 50], this implied that the OMAS cannot discriminate between a clinically important change from a measurement error of the instrument when the scores are between these two values. The quality was rated very low due to considerable risk of bias, but it still signifies the importance of reporting the measurement error and MIC.

In the assessment of subjective outcome measures such as PROMs, one can hardly declare an instrument to accommodate nearly perfect validity and reliability, hence the reluctant use of the word “gold standard”. In situations where PROMs are compared to each other, hypotheses are formed based on the assumed construct of each instrument while simultaneously acknowledging the current evidence on the comparator instruments’ measurement properties. The hypothesis testing of construct validity perhaps provides the least information regarding the validity of the application of an instrument since the method depends on the measurement properties of the comparator instruments and on the inquiring hypotheses postulated by the reviewers. However, acquiring evidence on this measurement property is a continuous process, and with growing empirical evidence, demonstration of construct validity is achievable through the process of probing hypotheses. In the current review, the OMAS was subject to the most hypothesis testing, with nine articles of varying methodological quality assessing construct validity, resulting in 75% confirmed hypotheses. The LEFS also had multiple studies of at least adequate methodological quality assessing construct validity with hypothesis testing, resulting in 87% confirmed hypotheses.

### Limitations

When methodologically adequate studies are missing in the assessment of content validity, the reviewers’ rating remained the only rating. Depending on the reviewers’ level of knowledge and experience, this can introduce bias in the assessment. Likewise, for the definition of hypothesis testing for construct validity, the categorization of expected correlations was discussed and agreed upon within the review team, but this might differ for other reviewers.

Seven articles were not included by the main search. Four of the articles did not include the word “fracture” in their title, abstract or keywords [34, 35, 37, 39]. They were also not indexed with subject headings for ankle fractures. The remaining three articles were excluded due to lack of terms or phrases found in the PROM-inclusion filter developed by the University of Oxford [33, 36, 38].

### Conclusion

None of the PROMs included in this study received a category A recommendation due to lack of evidence on sufficient content validity and internal consistency. In addition, none of the PROMs had good evidence on an insufficient measurement property, leaving category C empty. Therefore, all PROMs included in this review were assigned to category B. Due to the lack of PROMs in category A, the OMAS, SEFAS and A-FORM received a temporary recommendation of use for evaluative purposes in the ankle fracture population pending additional evidence. Further research should focus on conducting high quality content validity studies for the PROMs used in this context. There is also a significant need for more empirical evidence on the remaining measurement properties of the A-FORM.

## Supplementary Information

Below is the link to the electronic supplementary material.

Supplementary file1 (PDF 229 KB)

Supplementary file2 (PDF 85 KB)

Supplementary file3 (PDF 104 KB)

Supplementary file4 (PDF 167 KB)

Supplementary file5 (PDF 226 KB)

Supplementary file6 (PDF 95 KB)
