# Supplementary material for: Ankle fractures: a systematic review of patient-reported outcome measures and their measurement properties
Source: Qual Life Res. 2022 Jun 18;32(1):27–45. doi: 10.1007/s11136-022-03166-3 (PMC9829578; doi:10.1007/s11136-022-03166-3)
Supplement: Supplementary file 1 — Supplementary file1 (PDF 229 KB) [file 11136_2022_3166_MOESM1_ESM.pdf]

Article title

Ankle Fractures: A Systematic Review of Patient Reported Outcome Measures and their measurement properties

Journal name

Quality of Life Research

Author information

Michael Quan Nguyen[1,2], Ingvild Dalen[2,3], Marjolein Memelink Iversen[4,5], Knut Harboe[1,6], Aksel Paulsen[1,7]

[1]Department of Orthopedic Surgery, Stavanger University Hospital, Helse Stavanger HF.
[2]Department of Quality and Health Technology, Faculty of Health Sciences, University of Stavanger.
[3]Department of Research, Stavanger University Hospital, Helse Stavanger HF.
[4]Centre on Patient-reported Outcomes, Department of Research and Development,
Haukeland University Hospital, Helse Bergen HF.
[5]Department of Health and Caring Sciences, Faculty of Health and Social Sciences, Western Norway University of Applied Sciences.
[6]Department of Clinical Medicine, Faculty of Medicine, University of Bergen.
[7]Department of Public Health, Faculty of Health Sciences, University of Stavanger.

Corresponding author:

Michael Quan Nguyen
E-mail: n.michael.quan@gmail.com
ORCID: 0000-0003-0270-9518

Online Resource 1 Search strategies

A literature search was performed in Medline, EMBASE and CINAHL. The initial search included articles from the inception of the databases to the 8th of February 2019, and an updated search, from the end date of the initial search to the 6th of July 2021. A PROM inclusion filter developed by the University of Oxford [1] was applied and supplemented with the MeSH term “patient reported outcome measures” in Medline, the subject heading “patient-reported outcome” in EMBASE, and a translation of the filter was made for CINAHL. A validated sensitive search filter for measurement properties by Terwee et al. [2] was translated and applied to all three databases. The exclusion filter was translated and applied in Medline. Subject headings for age groups were used to exclude results indexed with child and adolescent age groups only.

The initial search contained different terms for long bone fracture, including MeSH subject headings and terms from the thesauri in EMBASE and CINAHL. Terms for the acetabulum, scapula, glenoid and clavicle were added. A decision was made to restrict the review to a particular anatomic location to enhance the conciseness of the review, and the search was updated to include terms relating only to ankle fractures. Articles fulfilling the eligibility criteria for the current review on ankle fractures were extracted from the initial search.

Long-bone fractures and PROMs

Ovid Embase and Medline 08.02.19:

Federated search in:
Embase <1974 to 2019 February 07>
Ovid MEDLINE(R) and Epub Ahead of Print, In-Process & Other Non-Indexed Citations and Daily <1946 to February 07, 2019>

#	Searches	Results
**Search strategy for Medline:**		
1	ankle fractures/ or femoral fractures/ or hip fractures/ or femoral neck fractures/ or humeral fractures/ or intra-articular fractures/ or radius fractures/ or colles' fracture/ or shoulder fractures/ or tibial fractures/ or ulna fractures/ or monteggia's fracture/	108502
2	((ankle or femoral or femur or hip or humeral or humerus or glenohumeral or glenoid or radial or radius or colles* or tibia* or ulna* or monteggia* or overarm* or underarm* or thigh* or leg or legs or long-bone* or elbow* or upper extremity or limb or arm or shoulder or olecranon or wrist or antebrachi* or forearm or fibula* or lower extremity or knee or kneecap or patella* or crus or shin bone or malleol* or supracondylar or clavicle or clavicula* or joint or talus or acetabul* or greater tronchanter or lesser trochanter or metacarpal or scaphoid or hand or foot or calcane* or phalanx or phalangeal or scapula or metatarsal or finger or talar or heel or hindfoot or midfoot or lisfranc or forefoot or toe) adj2 fracture*).ti,ab,kw.	156860
3	1 or 2	203733
4	Infant/ or Child/ or Adolescent/ or Minors/ or Child, Preschool/ or Infant, Newborn/	6346178
5	adult/ or aged/ or "aged, 80 and over"/ or frail elderly/ or middle aged/ or young adult/	14057538
6	4 and 5	2689878
7	4 not 6	3656300
8	3 not 7	184676
9	(HR-PRO or HRPRO or HRQL or HRQoL or QL or QoL).ti,ab. or quality of life.mp. or (health index* or health indices or health profile*).ti,ab. or health status.mp. or ((patient or self or child or parent or carer or proxy) adj (appraisal* or appraised or report or reported or reporting or rated or rating* or based or assessed or assessment*)).ti,ab. or ((disability or function or functional or functions or subjective or utility or utilities or wellbeing or well being) adj2 (index or indices or instrument or instruments or measure or measures or questionnaire* or profile or profiles or scale or scales or score or scores or status or survey or surveys)).ti,ab.	1646378
10	patient reported outcome measures/	13662
11	9 or 10	1648113
12	8 and 11	11804
13	(addresses or biography or case reports or comment or directory or editorial or festschrift or interview or lectures or legal cases or legislation or letter or news or newspaper article or patient education handout or popular works or congresses or consensus development conference or consensus development conference, nih or practice guideline).pt. not (exp animals/ not humans.sh.)	4075287

14	12 not 13	11580
15	14 use ppez	4865
16	(instrumentation or methods).fs. or (Validation Studies or Comparative Study).pt. or Psychometrics/ or psychometr*.ti,ab,kw. or (clinimetr* or clinometr*).mp. or exp "Outcome Assessment (Health Care)"/ or outcome assessment.ti,ab,kw. or outcome measure*.mp. or Observer Variation/ or observer variation.ti,ab,kw. or exp Health Status Indicators/ or exp "Reproducibility of Results"/ or reproducib*.ti,ab,kw. or Discriminant Analysis/ or (reliab* or unreliab* or valid* or coefficient or homogeneity or homogeneous or internal consistency).ti,ab,kw. or (cronbach* and (alpha or alphas)).ti,ab,kw. or (item and (correlation* or selection* or reduction*)).ti,ab,kw. or (agreement or precision or imprecision or precise values).mp. or (test-retest or (test and retest)).ti,ab,kw. or (reliab* and (test or retest)).ti,ab,kw. or (stability or interrater or inter-rater or intrarater or intra-rater or intertester or inter-tester or intratester or intra-tester or interobserver or inter-observer or intraobserver or intra-observer or intertechnician or inter-technician or intratechnician or intra-technician or interexaminer or inter-examiner or intraexaminer or intra-examiner or interassay or inter-assay or intraassay or intra-assay or interindividual or inter-individual or intraindividual or intra-individual or interparticipant or inter-participant or intraparticipant or intra-participant or kappa or kappa's or kappas).ti,ab,kw. or repeatab*.mp. or ((replicab* or repeated) and (measure or measures or findings or result or results or test or tests)).mp. or (generaliza* or generalisa* or concordance).ti,ab,kw. or (intraclass and correlation*).ti,ab,kw. or (discriminative or known group or factor analysis or factor analyses or factor structure* or dimension* or subscale*).ti,ab,kw. or (multitrait and scaling and (analysis or analyses)).ti,ab,kw. or (item discriminant or interscale correlation* or error or errors or individual variability or interval variability or rate variability).ti,ab,kw. or (variability and (analysis or values)).ti,ab,kw. or (uncertainty and (measurement or measuring)).ti,ab,kw. or (sensitiv* or responsive*).ti,ab,kw. or (limit and detection).ti,ab,kw. or (minimal detectable concentration or interpretab*).ti,ab,kw. or ((minimal or minimally or clinical or clinically) and (important or significant or detectable) and (change or difference)).ti,ab,kw. or (small* and (real or detectable) and (change or difference)).ti,ab,kw. or (meaningful change or ceiling effect or floor effect or Item response model or IRT or Rasch or Differential item functioning or DIF or computer adaptive testing or item bank or cross-cultural equivalence).ti,ab,kw.	14837891
17	16 use ppez	8914774
18	15 and 17	3473
**Search strategy for Embase:**		
19	clavicle fracture/ or joint fracture/ or ankle fracture/ or malleolus fracture/ or lateral malleolar fracture/ or medial malleolar fracture/ or talus fracture/ or elbow fracture/ or humeral supracondylar fracture/ or olecranon fracture/ or radius head fracture/ or hip fracture/ or acetabulum fracture/ or femoral head fracture/ or femoral neck fracture/ or femoral intertrochanteric fracture/ or femur pertochanteric fracture/ or femur subtrochanteric fracture/ or femur trochanteric fracture/ or fracture of greater trochanter/ or fracture of lesser trochanter/ or knee fracture/ or shoulder fracture/ or proximal humerus fracture/ or humeral head fracture/ or humeral neck fracture/ or wrist fracture/ or distal radius fracture/ or Colles fracture/ or metacarpal bone fracture/ or limb fracture/ or arm fracture/ or forearm fracture/ or radius fracture/ or proximal radius fracture/ or radius shaft fracture/ or ulna fracture/ or monteggia fracture/ or scaphoid fracture/ or hand fracture/ or distal humeral fracture/ or humerus shaft fracture/ or leg fracture/ or femur fracture/ or distal femur fracture/ or femur shaft fracture/ or proximal femur fracture/ or fibula fracture/ or distal fibula fracture/ or fibula shaft fracture/ or proximal fibula fracture/ or foot fracture/ or calcaneus fracture/ or patella fracture/ or tibia fracture/ or distal tibia fracture/ or proximal tibia fracture/ or tibial plateau fracture/ or tibia shaft fracture/ or phalanx fracture/ or scapula fracture/	133069
20	((ankle or femoral or femur or hip or humeral or humerus or glenohumeral or glenoid or radial or radius or colles* or tibia* or ulna* or monteggia* or overarm* or underarm* or thigh* or leg or legs or long-bone* or elbow* or upper extremity or limb or arm or shoulder or olecranon or wrist or antebrachi* or forearm or fibula* or lower extremity or knee or kneecap or patella* or crus or shin bone or malleol* or supracondylar or clavicle or clavicula* or joint or talus or acetabul* or greater tronchanter or lesser trochanter or metacarpal or scaphoid or hand or foot or calcane* or phalanx or	156860

	phalangeal or scapula or metatarsal or finger or talar or heel or hindfoot or midfoot or lisfranc or forefoot or toe) adj2 fracture*).ti,ab,kw.	
21	19 or 20	207899
22	adolescent/ or juvenile/ or child/ or preschool child/ or school child/ or toddler/ or infant/ or newborn/ or baby/	6466726
23	adult/ or young adult/ or middle aged/ or aged/ or frail elderly/ or very elderly/	14030696
24	22 and 23	2711020
25	22 not 24	3755706
26	21 not 25	189406
27	(HR-PRO or HRPRO or HRQL or HRQoL or QL or QoL).ti,ab. or quality of life.mp. or (health index* or health indices or health profile*).ti,ab. or health status.mp. or ((patient or self or child or parent or carer or proxy) adj (appraisal* or appraised or report or reported or reporting or rated or rating* or based or assessed or assessment*)).ti,ab. or ((disability or function or functional or functions or subjective or utility or utilities or wellbeing or well being) adj2 (index or indices or instrument or instruments or measure or measures or questionnaire* or profile or profiles or scale or scales or score or scores or status or survey or surveys)).ti,ab.	1646378
28	patient-reported outcome/	15654
29	27 or 28	1648287
30	26 and 29	12812
31	limit 30 to embase [Limit not valid in Ovid MEDLINE(R),Ovid MEDLINE(R) Daily Update,Ovid MEDLINE(R) PubMed not MEDLINE,Ovid MEDLINE(R) In-Process,Ovid MEDLINE(R) Publisher; records were retained]	10580
32	31 use oemezd	5675
33	intermethod comparison/ or exp data collection method/ or validation study/ or feasibility study/ or pilot study/ or exp psychometry/ or psychometr*.ti,ab,kw. or (clinimetr* or clinometr*).mp. or outcome assessment/ or outcome assessment.ti,ab,kw. or outcome measure*.mp. or Observer Variation/ or observer variation.ti,ab,kw. or exp health status indicator/ or reproducibility/ or reproducib*.ti,ab,kw. or Discriminant Analysis/ or exp validity/ or (reliab* or unreliab* or valid* or coefficient or homogeneity or homogeneous or internal consistency).ti,ab,kw. or (cronbach* and (alpha or alphas)).ti,ab,kw. or (item and (correlation* or selection* or reduction*)).ti,ab,kw. or (agreement or precision or imprecision or precise values).mp. or (test-retest or (test and retest)).ti,ab,kw. or (reliab* and (test or retest)).ti,ab,kw. or (stability or interrater or inter-rater or intrarater or intra-rater or intertester or inter-tester or intratester or intra-tester or interobserver or inter-observer or intraobserver or intra-observer or intertechnician or inter-technician or intratechnician or intra-technician or interexaminer or inter-examiner or intraexaminer or intra-examiner or interassay or inter-assay or intraassay or intra-assay or interindividual or inter-individual or intraindividual or intra-individual or interparticipant or inter-participant or intraparticipant or intra-participant or kappa or kappa's or kappas).ti,ab,kw. or repeatab*.mp. or ((replicab* or repeated) and (measure or measures or findings or result or results or test or tests)).mp. or (generaliza* or generalisa* or concordance).ti,ab,kw. or (intraclass and correlation*).ti,ab,kw. or (discriminative or known group or factor analysis or factor analyses or factor structure* or dimension* or subscale*).ti,ab,kw. or (multitrait and scaling and (analysis or analyses)).ti,ab,kw. or (item discriminant or interscale correlation* or error or errors or individual variability or interval variability or rate variability).ti,ab,kw. or (variability and (analysis or values)).ti,ab,kw. or (uncertainty and (measurement or measuring)).ti,ab,kw. or (sensitiv* or responsive*).ti,ab,kw. or (limit and detection).ti,ab,kw. or (minimal detectable concentration or interpretab*).ti,ab,kw. or ((minimal or minimally or clinical or clinically) and (important or significant or detectable) and (change or difference)).ti,ab,kw. or (small* and (real or detectable) and (change or difference)).ti,ab,kw. or (meaningful change or ceiling effect or floor effect or Item response model or IRT or Rasch or Differential item functioning or DIF or computer adaptive testing or item bank or cross-cultural equivalence).ti,ab,kw.	12971509
34	33 use oemezd	6829880
35	32 and 34	2906
36	18 or 35	6379
37	limit 36 to yr="2013 -Current"	3274

38	36 not 37	3105
39	remove duplicates from 37	2386
40	remove duplicates from 38	2334
41	39 or 40	4720
42	41 use ppez	1863
43	41 use oemezd	2857

The results set for search line 36 was too big for deduplication in Ovid, which is why it is split in search lines 37 and 38.
Ppez = Ovid MEDLINE(R) and Epub Ahead of Print, In-Process & Other Non-Indexed Citations and Daily, 1946 to present
Oemezd = Embase, 1974 to present, updated daily

Cinahl 08.02.19:

Interface - EBSCOhost Research Databases
Search Screen - Advanced Search
Database - CINAHL with Full Text
Search modes - Boolean/Phrase

**Search ID#**	**Search Terms**	**Results**
S12	S10 AND S11	326
S11	(MH "Validation Studies") OR (MH "Comparative Studies") OR (MH "Psychometrics") OR (TI psychometr* OR AB psychometr*) OR clinimetr* OR clinometr* OR (MH "Outcome Assessment") OR (TI "outcome assessment" OR AB "outcome assessment") OR "outcome measure*" OR "observer variation" OR (MH "Health Status Indicators") OR (MH "Reproducibility of Results") OR (TI reproducib* OR AB reproducib*) OR (MH "Discriminant Analysis") OR (MH "Content Validity+") OR (MH "Criterion-Related Validity+") OR (MH "Construct Validity+") OR (MH "Reliability+") OR (TI reliab* OR AB reliab*) OR (TI unreliab* OR AB unreliab*) OR (TI valid* OR AB valid*) OR (TI coefficient OR AB coefficient) OR (TI homogeneity OR AB homogeneity) OR (TI homogeneous OR AB homogeneous) OR (TI "internal consistency" OR AB "internal consistency") OR ((TI cronbach* OR AB cronbach*) AND (TI alpha OR AB alpha OR TI alphas OR AB alphas)) OR ((TI item OR AB item) AND (TI correlation* OR AB correlation* OR TI selection* OR AB selection* OR TI reduction* OR AB reduction*)) OR agreement OR precision OR imprecision OR "precise values" OR (TI test-retest OR AB test-retest) OR ((TI test OR AB test) AND (TI retest OR AB retest)) OR ((TI reliab* OR AB reliab*) AND (TI test OR AB test OR TI retest or AB retest)) OR (TI stability OR AB stability) OR (TI interrater OR AB interrater) OR (TI inter-rater OR AB inter-rater) OR (TI intrarater OR AB intrarater) OR (TI intra-rater OR AB intra-rater) OR (TI intertester OR AB intertester) OR (TI inter-tester OR AB inter-tester) OR (TI intratester OR AB intratester) OR (TI intra-tester OR AB intra-tester) OR (TI interobserver OR AB interobserver) OR (TI inter-observer OR AB inter-observer) OR (TI intraobserver OR AB intraobserver) OR (TI intra-observer OR AB intra-observer) OR (TI intertechnician OR AB intertechnician) OR (TI inter-technician OR AB inter-technician) OR (TI intratechnician OR AB intratechnician) OR (TI intra-technician OR AB intra-technician) OR (TI interexaminer OR AB interexaminer) OR (TI inter-examiner OR AB inter-examiner) OR (TI intraexaminer OR AB intraexaminer) OR (TI intra-examiner OR AB intra-examiner) OR (TI intra-examiner OR AB intraexaminer) OR (TI interassay OR AB interassay) OR (TI inter-assay OR AB inter-assay) OR (TI intraassay OR AB intraassay) OR (TI intra-assay OR AB intra-assay) OR (TI interindividual OR AB interindividual) OR (TI inter-individual OR AB inter-individual) OR (TI intraindividual OR AB intraindividual) OR (TI intra-individual OR AB intra-individual) OR (TI interparticipant OR AB interparticipant) OR (TI inter-participant OR AB inter-participant) OR (TI intraparticipant OR AB intraparticipant) OR (TI intra-participant OR AB intra-participant) OR (TI kappa OR AB kappa) OR (TI kappa's OR AB kappa's) OR (TI kappas OR AB kappas) OR repeatab* OR ((replicab* OR repeated) AND (measure OR measures OR findings OR result OR results OR test OR tests)) OR TI generaliza* OR AB generaliza* OR TI generalisa* OR AB generalisa* OR TI concordance OR AB concordance OR ((TI intraclass OR AB intraclass) AND (TI	971,490

	correlation* OR AB correlation*)) OR TI discriminative OR AB discriminative OR TI "known group" OR AB "known group" OR TI "factor analysis" OR AB "factor analysis" OR TI "factor analyses" OR AB "factor analyses" OR TI dimension* OR AB dimension* OR TI subscale* OR AB subscale* OR ((TI multitrait OR AB multitrait) AND (TI scaling OR AB scaling) AND (TI analysis OR AB analysis OR TI analyses OR AB analyses)) OR TI "item discriminant" OR AB "item discriminant" OR TI "interscale correlation*" OR AB "interscale correlation*" OR TI error OR AB error OR TI errors OR AB errors OR TI "individual variability" OR AB "individual variability" OR TI "interval variability" OR AB "interval variability" OR TI "rate variability" OR AB "rate variability" OR ((TI variability OR AB variability) AND (TI analysis OR AB analysis OR TI values OR AB values) OR (TI uncertainty OR AB uncertainty) AND (TI measurement OR AB measurement OR TI measuring OR AB measuring)) OR (MH "Measurement Error+") OR (TI sensitiv* OR AB sensitiv* OR TI responsive* OR AB responsive*) OR ((TI limit OR AB limit) AND (TI detection OR AB detection)) OR "minimal detectable concentration" OR (TI interpretab* or AB interpretab*) OR ((minimal OR minimally OR clinical OR clinically) AND (important OR significant OR detectable) AND (change OR difference)) OR ((TI small* OR AB small*) AND (TI real OR AB real OR TI detectable OR AB detectable) AND (TI change OR AB change OR TI difference OR AB difference)) OR "meaningful change" OR "ceiling effect" OR "floor effect" OR "Item response model" OR TI IRT OR AB IRT OR TI Rasch OR AB Rasch OR "Differential item functioning" OR TI DIF OR AB DIF OR "computer adaptive testing" OR "item bank" OR "cross-cultural equivalence"		
S10	S8 AND S9	Limiters - Exclude MEDLINE records	820
S9	"HR-PRO" or "HRPRO" or "HRQL" or "HRQoL" or "QL" or "QoL" or "quality of life" or "health index*" or "health indices" or "health profile*" or "health status" or ((patient or self or child or parent or carer or proxy) W0 (appraisal* or appraised or report or reported or reporting or rated or rating* or based or assessed or assessment*)) or ((disability or function or functional or functions or subjective or utility or utilities or wellbeing or "well being") N1 (index or indices or instrument or instruments or measure or measures or questionnaire* or profile or profiles or scale or scales or score or scores or status or survey or surveys))		374,984
S8	S3 not S7		28,911
S7	S4 not S6		496,197
S6	S4 AND S5		314,745
S5	(MH "Adult") OR (MH "Aged") OR (MH "Aged, 80 and Over") OR (MH "Frail Elderly") OR (MH "Middle Age") OR (MH "Young Adult")		1,517,289
S4	(MH "Child") OR (MH "Child, Preschool") OR (MH "Minors (Legal)") OR (MH "Infant, Newborn") OR (MH "Infant") OR (MH "Adolescence")		810,942
S3	S1 OR S2		31,736
S2	(ankle or femoral or femur or hip or humeral or humerus or glenohumeral or glenoid or radial or radius or colles* or tibia* or ulna* or monteggia* or overarm* or underarm* or thigh* or leg or legs or long-bone* or elbow* or "upper extremity" or limb or arm or shoulder or olecranon or wrist or antebrachi* or forearm or fibula* or "lower extremity" or knee or kneecap or patella* or crus or "shin bone" or malleol* or supracondylar or clavicle or clavicula* or joint or talus or acetabul* or "greater tronchanter" or "lesser trochanter" or metacarpal or scaphoid or hand or foot or calcane* or phalanx or phalangeal or scapula or metatarsal or finger or talar or heel or hindfoot or midfoot or lisfranc or forefoot or toe) N1 fracture*		31,687
S1	(MH "Ankle Fractures") or (MH "Elbow Fractures") or (MH "Femoral Fractures") or (MH "Hip Fractures") or (MH "Fibula Fractures") or (MH "Foot Fractures") or (MH "Calcaneus Fractures") or (MH "Metatarsal Fractures") or (MH "Hand Fractures") or (MH "Finger Fractures") or (MH "Metacarpal Fractures") or (MH "Humeral Fractures") or (MH "Knee Fractures") or (MH "Tibial Plateau Fracture") or (MH "Radius Fractures") or (MH "Shoulder Fractures") or (MH "Clavicle Fractures") or (MH "Tibial Fractures") or (MH "Tibial Plateau Fracture") or (MH "Ulna Fractures") or (MH "Ulna Fracture, Proximal") or (MH "Wrist Fractures") or (MH "Carpal Fractures")		22,719

Ankle fractures and PROMs

Ovid Embase and Medline 05.07.21:

Federated search in:
Embase <1974 to 2021 July 06>
Ovid MEDLINE(R) ALL <1946 to July 06, 2021>

#	Searches	Results
**Search strategy for Medline:**		
1	ankle fractures/ or tibial fractures/	29749
2	((ankle or tibia* or fibula* or malleol*) adj2 fracture*).ti,ab,kw.	28902
3	1 or 2	44135
4	Infant/ or Child/ or Adolescent/ or Minors/ or Child, Preschool/ or Infant, Newborn/	7120221
5	adult/ or aged/ or "aged, 80 and over"/ or frail elderly/ or middle aged/ or young adult/	16644812
6	4 and 5	3019539
7	4 not 6	4100682
8	3 not 7	40067
9	(HR-PRO or HRPRO or HRQL or HRQoL or QL or QoL).ti,ab. or quality of life.mp. or (health index* or health indices or health profile*).ti,ab. or health status.mp. or ((patient or self or child or parent or carer or proxy) adj (appraisal* or appraised or report or reported or reporting or rated or rating* or based or assessed or assessment*)).ti,ab. or ((disability or function or functional or functions or subjective or utility or utilities or wellbeing or well being) adj2 (index or indices or instrument or instruments or measure or measures or questionnaire* or profile or profiles or scale or scales or score or scores or status or survey or surveys)).ti,ab.	2056351
10	patient reported outcome measures/	37756
11	9 or 10	2060886
12	8 and 11	2308
13	(addresses or biography or case reports or comment or directory or editorial or festschrift or interview or lectures or legal cases or legislation or letter or news or newspaper article or patient education handout or popular works or congresses or consensus development conference or consensus development conference, nih or practice guideline).pt. not (exp animals/ not humans.sh.)	4655130
14	12 not 13	2254
15	14 use medall	991
16	(instrumentation or methods).fs. or (Validation Studies or Comparative Study).pt. or Psychometrics/ or psychometr*.ti,ab,kw. or (clinimetr* or clinometr*).mp. or exp "Outcome Assessment (Health Care)"/ or outcome assessment.ti,ab,kw. or outcome measure*.mp. or Observer Variation/ or observer variation.ti,ab,kw. or exp Health Status Indicators/ or exp "Reproducibility of Results"/ or reproducib*.ti,ab,kw. or Discriminant Analysis/ or (reliab* or unreliab* or valid* or coefficient or homogeneity or homogeneous or internal consistency).ti,ab,kw. or (cronbach* and (alpha or alphas)).ti,ab,kw. or (item and (correlation* or selection* or reduction*)).ti,ab,kw. or (agreement or precision or imprecision or precise values).mp. or (test-retest or (test and retest)).ti,ab,kw. or (reliab* and (test or retest)).ti,ab,kw. or (stability or interrater or inter-rater or intrarater or intra-rater or intertester or inter-tester or intratester or intra-tester or interobserver or inter-observer or intraobserver or intra-observer or intertechnician or inter-technician or intratechnician or intra-technician or interexaminer or inter-examiner or intraexaminer or intra-examiner or interassay or inter-assay or intraassay or intra-assay or interindividual or inter-individual or intraindividual or intra-individual or interparticipant or inter-participant or intraparticipant or intra-participant or kappa or kappa's or kappas).ti,ab,kw. or repeatab*.mp. or ((replicab* or repeated) and (measure or measures or findings or result or results or test or tests)).mp. or (generaliza* or generalisa* or concordance).ti,ab,kw. or (intraclass and correlation*).ti,ab,kw. or (discriminative or known group or factor analysis or factor analyses or factor structure* or dimension* or	17406881

	subscale*).ti,ab,kw. or (multitrait and scaling and (analysis or analyses)).ti,ab,kw. or (item discriminant or interscale correlation* or error or errors or individual variability or interval variability or rate variability).ti,ab,kw. or (variability and (analysis or values)).ti,ab,kw. or (uncertainty and (measurement or measuring)).ti,ab,kw. or (sensitiv* or responsive*).ti,ab,kw. or (limit and detection).ti,ab,kw. or (minimal detectable concentration or interpretab*).ti,ab,kw. or ((minimal or minimally or clinical or clinically) and (important or significant or detectable) and (change or difference)).ti,ab,kw. or (small* and (real or detectable) and (change or difference)).ti,ab,kw. or (meaningful change or ceiling effect or floor effect or Item response model or IRT or Rasch or Differential item functioning or DIF or computer adaptive testing or item bank or cross-cultural equivalence).ti,ab,kw.	
17	16 use medall	10235347
18	15 and 17	827
**Search strategy for Embase:**		
19	ankle fracture/ or malleolus fracture/ or lateral malleolar fracture/ or medial malleolar fracture/ or fibula fracture/ or distal fibula fracture/ or fibula shaft fracture/ or proximal fibula fracture/ or tibia fracture/ or distal tibia fracture/	22465
20	((ankle or tibia* or fibula* or malleol*) adj2 fracture*).ti,ab,kw.	28810
21	19 or 20	39362
22	adolescent/ or juvenile/ or child/ or preschool child/ or school child/ or toddler/ or infant/ or newborn/ or baby/	7236340
23	adult/ or young adult/ or middle aged/ or aged/ or frail elderly/ or very elderly/	16609392
24	22 and 23	3041069
25	22 not 24	4195271
26	21 not 25	36105
27	(HR-PRO or HRPRO or HRQL or HRQoL or QL or QoL).ti,ab. or quality of life.mp. or (health index* or health indices or health profile*).ti,ab. or health status.mp. or ((patient or self or child or parent or carer or proxy) adj (appraisal* or appraised or report or reported or reporting or rated or rating* or based or assessed or assessment*)).ti,ab. or ((disability or function or functional or functions or subjective or utility or utilities or wellbeing or well being) adj2 (index or indices or instrument or instruments or measure or measures or questionnaire* or profile or profiles or scale or scales or score or scores or status or survey or surveys)).ti,ab.	2056351
28	patient-reported outcome/	40389
29	27 or 28	2061124
30	26 and 29	2386
31	limit 30 to embase [Limit not valid in Ovid MEDLINE(R),Ovid MEDLINE(R) Daily Update,Ovid MEDLINE(R) PubMed not MEDLINE,Ovid MEDLINE(R) In-Process,Ovid MEDLINE(R) Publisher; records were retained]	2051
32	31 use oemezd	1140
33	intermethod comparison/ or exp data collection method/ or validation study/ or feasibility study/ or pilot study/ or exp psychometry/ or psychometr*.ti,ab,kw. or (clinimetr* or clinometr*).mp. or outcome assessment/ or outcome assessment.ti,ab,kw. or outcome measure*.mp. or Observer Variation/ or observer variation.ti,ab,kw. or exp health status indicator/ or reproducibility/ or reproducib*.ti,ab,kw. or Discriminant Analysis/ or exp validity/ or (reliab* or unreliab* or valid* or coefficient or homogeneity or homogeneous or internal consistency).ti,ab,kw. or (cronbach* and (alpha or alphas)).ti,ab,kw. or (item and (correlation* or selection* or reduction*)).ti,ab,kw. or (agreement or precision or imprecision or precise values).mp. or (test-retest or (test and retest)).ti,ab,kw. or (reliab* and (test or retest)).ti,ab,kw. or (stability or interrater or inter-rater or intrarater or intra-rater or intertester or inter-tester or intratester or intra-tester or interobserver or inter-observer or intraobserver or intra-observer or intertechnician or inter-technician or intratechnician or intra-technician or interexaminer or inter-examiner or intraexaminer or intra-examiner or interassay or inter-assay or intraassay or intra-assay or interindividual or inter-individual or intraindividual or intra-individual or interparticipant or	15519351

	inter-participant or intraparticipant or intra-participant or kappa or kappa's or kappas).ti,ab,kw. or repeatab*.mp. or ((replicab* or repeated) and (measure or measures or findings or result or results or test or tests)).mp. or (generaliza* or generalisa* or concordance).ti,ab,kw. or (intraclass and correlation*).ti,ab,kw. or (discriminative or known group or factor analysis or factor analyses or factor structure* or dimension* or subscale*).ti,ab,kw. or (multitrait and scaling and (analysis or analyses)).ti,ab,kw. or (item discriminant or interscale correlation* or error or errors or individual variability or interval variability or rate variability).ti,ab,kw. or (variability and (analysis or values)).ti,ab,kw. or (uncertainty and (measurement or measuring)).ti,ab,kw. or (sensitiv* or responsive*).ti,ab,kw. or (limit and detection).ti,ab,kw. or (minimal detectable concentration or interpretab*).ti,ab,kw. or ((minimal or minimally or clinical or clinically) and (important or significant or detectable) and (change or difference)).ti,ab,kw. or (small* and (real or detectable) and (change or difference)).ti,ab,kw. or (meaningful change or ceiling effect or floor effect or Item response model or IRT or Rasch or Differential item functioning or DIF or computer adaptive testing or item bank or cross-cultural equivalence).ti,ab,kw.	
34	33 use oemezd	8280481
35	32 and 34	710
36	18 or 35	1537
37	remove duplicates from 36	1117
38	37 use medall	415
39	37 use oemezd	702

medall = Ovid MEDLINE(R) ALL, 1946 to daily update
oemezd = Embase, 1974 to present, updated daily

Cinahl 05.07.21:

Interface - EBSCOhost Research Databases
Search Screen - Advanced Search
Database - CINAHL with Full Text
Search modes - Boolean/Phrase

#	Query	Results
S12	S10 AND S11	97
S11	(MH "Validation Studies") OR (MH "Comparative Studies") OR (MH "Psychometrics") OR (TI psychometr* OR AB psychometr*) OR clinimetr* OR clinometr* OR (MH "Outcome Assessment") OR (TI "outcome assessment" OR AB "outcome assessment") OR "outcome measure*" OR "observer variation" OR (MH "Health Status Indicators") OR (MH "Reproducibility of Results") OR (TI reproducib* OR AB reproducib*) OR (MH "Discriminant Analysis") OR (MH "Content Validity+") OR (MH "Criterion-Related Validity+") OR (MH "Construct Validity+") OR (MH "Reliability+") OR (TI reliab* OR AB reliab*) OR (TI unreliab* OR AB unreliab*) OR (TI valid* OR AB valid*) OR (TI coefficient OR AB coefficient) OR (TI homogeneity OR AB homogeneity) OR (TI homogeneous OR AB homogeneous) OR (TI "internal consistency" OR AB "internal consistency") OR ((TI cronbach* OR AB cronbach*) AND (TI alpha OR AB alpha OR TI alphas OR AB alphas)) OR ((TI item OR AB item) AND (TI correlation* OR AB correlation* OR TI selection* OR AB selection* OR TI reduction* OR AB reduction*)) OR agreement OR precision OR imprecision OR "precise values" OR (TI test-retest OR AB test-retest) OR ((TI test OR AB test) AND (TI retest OR AB retest)) OR ((TI reliab* OR AB reliab*) AND (TI test OR AB test OR TI retest or AB retest)) OR (TI stability OR AB stability) OR (TI interrater OR AB interrater) OR (TI inter-rater OR AB inter-rater) OR (TI intrarater OR AB intrarater) OR (TI intra-rater OR AB intra-rater) OR (TI intertester OR AB intertester) OR (TI inter-tester OR AB inter-tester) OR (TI intratester OR AB intratester) OR (TI intra-tester OR AB intra-tester) OR (TI interobserver OR AB interobserver) OR (TI inter-observer OR AB inter-observer) OR (TI intraobserver OR AB intraobserver) OR (TI intra-observer OR AB intra-observer) OR (TI intertechnician OR AB intertechnician) OR (TI inter-technician OR AB inter-technician) OR (TI intratechnician OR AB intratechnician) OR (TI intra-technician OR AB intra-technician) OR (TI interexaminer OR AB interexaminer) OR (TI inter-examiner OR AB inter-examiner) OR (TI intraexaminer OR AB intraexaminer) OR (TI intra-examiner OR AB intra-examiner) OR (TI intra-examiner OR AB intraexaminer) OR (TI interassay OR AB interassay) OR (TI inter-assay OR AB inter-assay) OR (TI intraassay OR AB intraassay) OR (TI intra-assay OR AB intra-assay) OR (TI interindividual OR AB interindividual) OR (TI inter-individual OR AB inter-individual) OR (TI intraindividual OR AB intraindividual) OR (TI intra-individual OR AB intra-individual) OR (TI interparticipant OR AB interparticipant) OR (TI inter-participant OR AB inter-participant) OR (TI intraparticipant OR AB intraparticipant) OR (TI intra-participant OR AB intra-participant) OR (TI kappa OR AB kappa) OR (TI kappa's OR AB kappa's) OR (TI kappas OR AB kappas) OR repeatab* OR ((replicab* OR repeated) AND (measure OR measures OR findings OR result OR results OR test OR tests)) OR TI generaliza* OR AB generaliza* OR TI generalisa* OR AB generalisa* OR TI concordance OR AB concordance OR ((TI intraclass OR AB intraclass) AND (TI correlation* OR AB correlation*)) OR TI discriminative OR AB discriminative OR TI "known group" OR AB "known group" OR TI "factor analysis" OR AB "factor analysis" OR TI "factor analyses" OR AB "factor analyses" OR TI dimension* OR AB dimension* OR TI subscale* OR AB subscale* OR ((TI multitrait OR AB multitrait) AND (TI scaling OR AB scaling) AND (TI analysis OR AB analysis OR TI analyses OR AB analyses)) OR TI "item discriminant" OR AB "item discriminant" OR TI "interscale correlation*" OR AB "interscale correlation*" OR TI error OR AB error OR TI errors OR AB errors OR TI "individual variability" OR AB "individual variability" OR TI "interval variability" OR AB "interval variability" OR TI "rate variability" OR AB "rate variability" OR ((TI variability OR AB variability) AND (TI analysis OR AB analysis OR TI values OR AB values) OR (TI uncertainty OR AB uncertainty) AND (TI measurement OR AB measurement OR TI measuring OR AB measuring)) OR (MH "Measurement Error+") OR (TI sensitiv* OR AB sensitiv* OR TI responsive* OR AB responsive*) OR ((TI limit OR AB limit) AND (TI detection OR AB detection)) OR "minimal detectable concentration" OR (TI interpretab* or AB interpretab*	1,373,769

	) OR ((minimal OR minimally OR clinical OR clinically) AND (important OR significant OR detectable) AND (change OR difference)) OR ((TI small* OR AB small*) AND (TI real OR AB real OR TI detectable OR AB detectable) AND (TI change OR AB change OR TI difference OR AB difference)) OR "meaningful change" OR "ceiling effect" OR "floor effect" OR "Item response model" OR TI IRT OR AB IRT OR TI Rasch OR AB Rasch OR "Differential item functioning" OR TI DIF OR AB DIF OR "computer adaptive testing" OR "item bank" OR "cross-cultural equivalence"		
S10	S8 AND S9	Limiters - Exclude MEDLINE records	199
S9	"HR-PRO" or "HRPRO" or "HRQL" or "HRQoL" or "QL" or "QoL" or "quality of life" or "health index*" or "health indices" or "health profile*" or "health status" or ((patient or self or child or parent or carer or proxy) W0 (appraisal* or appraised or report or reported or reporting or rated or rating* or based or assessed or assessment*)) or ((disability or function or functional or functions or subjective or utility or utilities or wellbeing or "well being") N1 (index or indices or instrument or instruments or measure or measures or questionnaire* or profile or profiles or scale or scales or score or scores or status or survey or surveys))		503,625
S8	S3 not S7		6,968
S7	S4 not S6		613,161
S6	S4 AND S5		389,809
S5	(MH "Adult") OR (MH "Aged") OR (MH "Aged, 80 and Over") OR (MH "Frail Elderly") OR (MH "Middle Age") OR (MH "Young Adult")		1,893,020
S4	(MH "Child") OR (MH "Child, Preschool") OR (MH "Minors (Legal)") OR (MH "Infant, Newborn") OR (MH "Infant") OR (MH "Adolescence")		1,002,970
S3	S1 OR S2		7,647
S2	(ankle or tibia* or fibula* or malleol*) N1 fracture*		7,647
S1	(MH "Ankle Fractures") or (MH "Fibula Fractures") or (MH "Tibial Fractures")		5,702

References:

1 Mackintosh, A., Comabella, C. C. i., Hadi, M., Gibbons, E., Fitzpatrick, R., & Roberts, N. (2010). PROM group construct & instrument type filters February 2010.

2 Terwee, C. B., Jansma, E. P., Riphagen, II, & de Vet, H. C. (2009). Development of a methodological PubMed search filter for finding studies on measurement properties of measurement instruments. *Quality of Life Research, 18*(8), 1115-1123. doi:10.1007/s11136-009-9528-5
