# Supplementary material for: Ankle fractures: a systematic review of patient-reported outcome measures and their measurement properties
Source: Qual Life Res. 2022 Jun 18;32(1):27–45. doi: 10.1007/s11136-022-03166-3 (PMC9829578; doi:10.1007/s11136-022-03166-3)
Supplement: Supplementary file 2 — Supplementary file2 (PDF 85 KB) [file 11136_2022_3166_MOESM2_ESM.pdf]

Article title

Ankle Fractures: A Systematic Review of Patient Reported Outcome Measures and their measurement properties

Journal name

Quality of Life Research

Author information

Michael Quan Nguyen[1,2], Ingvild Dalen[2,3], Marjolein Memelink Iversen[4,5], Knut Harboe[1,6], Aksel Paulsen[1,7]

[1]Department of Orthopedic Surgery, Stavanger University Hospital, Helse Stavanger HF.
[2]Department of Quality and Health Technology, Faculty of Health Sciences, University of Stavanger.
[3]Department of Research, Stavanger University Hospital, Helse Stavanger HF.
[4]Centre on Patient-reported Outcomes, Department of Research and Development, Haukeland University Hospital, Helse Bergen HF.
[5]Department of Health and Caring Sciences, Faculty of Health and Social Sciences, Western Norway University of Applied Sciences.
[6]Department of Clinical Medicine, Faculty of Medicine, University of Bergen.
[7]Department of Public Health, Faculty of Health Sciences, University of Stavanger.

Corresponding author:

Michael Quan Nguyen
E-mail: n.michael.quan@gmail.com
ORCID: 0000-0003-0270-9518

Online Resource 2 Threshold categories for correlations and ground set of hypotheses for construct validity

Table 1 The threshold categories for correlations with comparator instruments

**Category**	**Correlation**
Weak	$r < 0.3$
Weak to moderate	$0.2 < r < 0.4$
Moderate	$0.3 < r < 0.7$
Moderate to high	$0.6 < r < 0.8$
High	$r > 0.7$

Table 2 The ground set of hypotheses for assessing construct validity

Hypothesis number	Hypothesis
1	Correlations with instruments measuring same construct should be at least moderate to high ($r > 0.6$)
2	Correlations with instruments measuring related constructs should be moderate ($0.3 < r < 0.7$)
3	Correlations with instruments measuring weakly related constructs should be weak to moderate ($0.2 < r < 0.4$)
