# Supplementary material for: Ankle fractures: a systematic review of patient-reported outcome measures and their measurement properties
Source: Qual Life Res. 2022 Jun 18;32(1):27–45. doi: 10.1007/s11136-022-03166-3 (PMC9829578; doi:10.1007/s11136-022-03166-3)
Supplement: Supplementary file 3 — Supplementary file3 (PDF 104 KB) [file 11136_2022_3166_MOESM3_ESM.pdf]

Article title

Ankle Fractures: A Systematic Review of Patient Reported Outcome Measures and their measurement properties

Journal name

Quality of Life Research

Author information

Michael Quan Nguyen[1,2], Ingvild Dalen[2,3], Marjolein Memelink Iversen[4,5], Knut Harboe[1,6], Aksel Paulsen[1,7]

[1]Department of Orthopedic Surgery, Stavanger University Hospital, Helse Stavanger HF.
[2]Department of Quality and Health Technology, Faculty of Health Sciences, University of Stavanger.
[3]Department of Research, Stavanger University Hospital, Helse Stavanger HF.
[4]Centre on Patient-reported Outcomes, Department of Research and Development,
Haukeland University Hospital, Helse Bergen HF.
[5]Department of Health and Caring Sciences, Faculty of Health and Social Sciences, Western Norway University of Applied Sciences.
[6]Department of Clinical Medicine, Faculty of Medicine, University of Bergen.
[7]Department of Public Health, Faculty of Health Sciences, University of Stavanger.

Corresponding author:

Michael Quan Nguyen
E-mail: n.michael.quan@gmail.com
ORCID: 0000-0003-0270-9518

Online Resource 3 Hypotheses for assessing construct validity of PROMs

**PROM under study**	**Reference**	**Comparator instrument/domains**	**Hypothesis number (direction)**
AAOS-FAOQ<br>Global foot and ankle scale	Zelle 2017	SF-36:<br>PCS<br>MCS	 2 (+)<br>3 (+)
AAOS-FAOQ<br>Shoe comfort scale	Zelle 2017	SF-36:<br>PCS<br>MCS	 2 (+)<br>3 (+)
LEFS	Garratt 2018, Lin 2009	OMAS	1 (+)
LEFS	Garratt 2018	SEFAS	1 (-)
LEFS	Garratt 2018	EQ-5D-3L domains:<br>- mobility<br>- self-care<br>- usual activities<br>- pain/discomfort<br>- anxiety/depression	 2 (-)<br>2 (-)<br>2 (-)<br>2 (-)<br>2 (-)
LEFS	Garratt 2018	EQ-5D-3L index	2 (-)
LEFS	Garratt 2018	SF-36 PF scale	2 (+)
LEFS	Repo 2017	15D domains:<br>- mobility<br>- usual activities<br>- discomfort and symptoms<br>- vitality	 1 (+)<br>1 (+)<br>2 (+)<br>2 (+)
MAQ-ADL	Greve 2018	FAAM-ADL	1 (+)
MAQ-ADL	Greve 2018	FAOS-ADL	1 (+)
MAQ-pain	Greve 2018	FAOS-pain	1 (+)
OMAS	Büker 2017, Nilsson 2013	GSRF	1 (-)
OMAS	Büker 2017, Nilsson 2013	FAOS domains:<br>- QoL<br>- symptoms/stiffness<br>- pain<br>- ADL<br>- sport	 1 (+)<br>1 (+)<br>1 (+)<br>1 (+)<br>1 (+)
OMAS	McKeown 2021	MOxFQ-index[1]	1 (-)
OMAS	McKeown 2021	MOxFQ-domains[1]:<br>- walking/standing<br>- pain<br>- social interaction	 1 (-)<br>1 (-)<br>1 (-)
OMAS	McKeown 2021	EQ-5D-5L domains:<br>- mobility<br>- self-care<br>- usual activities<br>- pain/discomfort<br>- anxiety/depression	 2 (-)<br>2 (-)<br>2 (-)<br>2 (-)<br>2 (-)
OMAS	McKeown 2021	EQ-5D-5L index	2 (+)

PROM under study	Reference	Comparator instrument/domains	Hypothesis number (direction)
OMAS	McKeown 2021	EQ-VAS	2 (+)
OMAS	McKeown 2021	DRI	2 (-)
OMAS	Garratt 2018	SEFAS	1 (-)
OMAS	Garratt 2018	LEFS	1 (+)
OMAS	Garratt 2018	EQ-5D-3L domains	
		- mobility	2 (-)
		- self-care	2 (-)
		- usual activities	2 (-)
		- pain/discomfort	2 (-)
		- anxiety/depression	2 (-)
OMAS	Garratt 2018	EQ-5D-3L index	2 (+)
OMAS	Garratt 2018	SF-36 PF	2 (+)
OMAS	Ponzer 1999	SF-36 domains:	
		- PF	2 (+)
		- role physical	2 (+)
		- pain	2 (+)
		- general health	2 (+)
		- social functioning	2 (+)
		- energy/fatigue	3 (+)
		- role emotional	3 (+)
		- mental health	3 (+)
OMAS	Ponzer 1999	VAS-physical health	2 (-)
OMAS	Ponzer 1999	VAS-mental health	3 (-)
OMAS	Turhan 2017	FAAM:	
		- ADL	1 (+)
		- sports	1 (+)
OMAS	Turhan 2017, Shah 2007	SF-12 PCS	2 (+)
OMAS	Shah 2007	SF-12 MCS	3 (+)
PROMIS LE CAT	Gausden 2018	FAOS domains:	
		- QoL	2 (+)
		- symptoms/stiffness	1 (+)
		- pain	1 (+)
		- ADL	1 (+)
		- Sport	1 (+)
PROMIS LE CAT	Gausden 2018	OMAS	1 (+)
PROMIS PF ver 1.2 CAT	Gausden 2018	FAOS domains:	
		- QoL	2 (+)
		- symptoms/stiffness	1 (+)
		- pain	1 (+)
		- ADL	1 (+)
		- sport	1 (+)
PROMIS PF ver 1.2 CAT	Gausden 2018	OMAS	1 (+)

**PROM under study**	**Reference**	**Comparator instrument/domains**	**Hypothesis number (direction)**
SEFAS	Garratt 2018	OMAS	1 (-)
SEFAS	Garratt 2018	LEFS	1 (-)
SEFAS	Garratt 2018	EQ-5D-3L domains<br>- mobility<br>- self-care<br>- usual activities<br>- pain/discomfort<br>- anxiety/depression	 2 (-)<br>2 (-)<br>2 (-)<br>2 (-)<br>2 (-)
SEFAS	Garratt 2018	EQ-5D-3L index	2 (-)
SEFAS	Garratt 2018	SF-36 PF	2 (-)
SMFA ADL	Obremskey 2007	SF-36 PF	1 (-)
SMFA bother index	Obremskey 2007	SF-36 domains:<br>- physical role limitations<br>- emotional role limitations	 2 (-)<br>2 (-)
SMFA dysfunction index	Obremskey 2007	SF-36 PF	2 (-)
SMFA emotional	Obremskey 2007	SF-36 domains:<br>- emotional role limitations<br>- mental health<br>- energy/fatigue	 2 (-)<br>2 (-)<br>2 (-)
SMFA mobility	Obremskey 2007	SF-36 PF	2 (-)
TEFTOM (only TOM part)	Suk 2013, Fang 2020	FAOS domains:<br>- QoL<br>- symptoms/stiffness<br>- pain<br>- ADL<br>- sport	 2 (+)<br>2 (+)<br>2 (+)<br>2 (+)<br>2 (+)
TEFTOM (only TOM part)	Suk 2013, Fang 2020	SF-36 PF	2 (+)
TEFTOM (only TOM part)	Suk 2013, Fang 2020	SF-36 PCS	2 (+)
TEFTOM (only TOM part)	Suk 2013, Fang 2020	AAOS	1 (+)
VAS-FA	Repo 2018	LEFS	1 (+)
VAS-FA	Repo 2018	15D index	2 (+)
WOMAC ver 3.0 ankle/foot (index score)	Ponkilainen 2019	LEFS	1 (-)
WOMAC ver 3.0 ankle/foot (index score)	Ponkilainen 2019	VAS-FA	1 (-)
WOMAC ver 3.0 ankle/foot (index score)	Ponkilainen 2019	15D index	2 (-)
WOMAC ver 3.0 ankle/foot (physical function subscale)	Ponkilainen 2019	LEFS	1 (-)

[1] foot and ankle

Abbreviations:
+ = positive correlation
- = negative correlation
AAOS-FAOQ = American Academy of Orthopaedic Surgeons Foot and Ankle Outcomes Questionnaire
ADL = activities of daily living
A-FORM = Ankle Fracture Outcome of Rehabilitation Measure
CAT = Computer Adaptive Test
DRI = Disability Rating Index
FAAM = Foot and Ankle Ability Measure
FAOS = Foot and Ankle Outcome Score
GSRF = Global Self-Rated Scale
LE = lower extremity
LEFS = Lower Extremity Functional Scale
MAQ = Munich Ankle Questionnaire
MCS = Mental Component Score
MOxFQ = Manchester-Oxford Foot Questionnaire
OMAS = Olerud-Molander Ankle Score
PCS = Physical Component Score
PF = physical function
PROMIS = Patient-Reported Outcomes Measurement Information System
QoL = quality of life
ROM = range of motion
SEFAS = Self-reported Foot and Ankle Score
SMFA = Short Musculoskeletal Function Assessment
TEFTOM = Trauma Expectation Factor Trauma Outcome Measure
VAS = Visual Analogue Scale
VAS-FA = Visual Analogue Scale Foot and Ankle
WOMAC = Western Ontario and McMaster Universities Osteoarthritis Index
