# Supplementary material for: Ankle fractures: a systematic review of patient-reported outcome measures and their measurement properties
Source: Qual Life Res. 2022 Jun 18;32(1):27–45. doi: 10.1007/s11136-022-03166-3 (PMC9829578; doi:10.1007/s11136-022-03166-3)
Supplement: Supplementary file 4 — Supplementary file4 (PDF 167 KB) [file 11136_2022_3166_MOESM4_ESM.pdf]

Article title

Ankle Fractures: A Systematic Review of Patient Reported Outcome Measures and their measurement properties

Journal name

Quality of Life Research

Author information

Michael Quan Nguyen[1,2], Ingvild Dalen[2,3], Marjolein Memelink Iversen[4,5], Knut Harboe[1,6], Aksel Paulsen[1,7]

[1]Department of Orthopedic Surgery, Stavanger University Hospital, Helse Stavanger HF.
[2]Department of Quality and Health Technology, Faculty of Health Sciences, University of Stavanger.
[3]Department of Research, Stavanger University Hospital, Helse Stavanger HF.
[4]Centre on Patient-reported Outcomes, Department of Research and Development, Haukeland University Hospital, Helse Bergen HF.
[5]Department of Health and Caring Sciences, Faculty of Health and Social Sciences, Western Norway University of Applied Sciences.
[6]Department of Clinical Medicine, Faculty of Medicine, University of Bergen.
[7]Department of Public Health, Faculty of Health Sciences, University of Stavanger.

Corresponding author:

Michael Quan Nguyen
E-mail: n.michael.quan@gmail.com
ORCID: 0000-0003-0270-9518

Online Resource 4 Characteristics of the comparator instruments

PROM	Reference	Construct	Target population	(Sub)scale(s) (number of items)	Response options	Range of scores/scoring
15D	Ponkilainen 2019, Repo 2018	General health	General, adults	15 domains / 15 items	1-5	Descriptive health profile or index based on utility weights: 0 (dead) - 1 (full "HrQoL")
American Academy of Orthopaedic Surgeons foot and ankle outcomes questionnaire, the global foot and ankle scale	Suk 2013, Fang 2020	Symtoms and functional status related to the foot and ankle	Foot and ankle problems	1 scale (the global foot and ankle scale) / 20 items. Pain (9) + funtion (6) + stiffness and swelling (2) + giving away (3). The shoe comfort scale: 5 items	Global foot and ankle scale: 1-3, 1-5, 1-6, 1-7.	0-100% (best)
Disability Rating Index	McKeown 2021	Physical function (disability)	Patients suffering from pain in the low back, neck and shoulders	12 items	VAS 0-100mm	Index: 0 (best) - 100 (worst)
EQ-5D-3L	Garratt 2018, Lash 2002	General health	Wide range of illnesses	5 domains (mobility, self-care, usual activities, pain/discomfort and anxiety/depression) / 5 items	1-3	Health profile or EQ-5D-index with value set
EQ-5D-5L	McKeown 2021	General health	Wide range of illnesses	5 domains (mobility, self-care, usual activities, pain/discomfort and anxiety/depression) / 5 items	1-5	Health profile or EQ-5D-index with value set

PROM	Reference	Construct	Target population	(Sub)scale(s) (number of items)	Response options	Range of scores/scoring
Foot and Ankle Outcome Score	Büker 2017, Nilsson 2013, Greve 2018, Suk 2013, Gausden 2018, Fang 2020	Symtoms and functional limitations related to the foot and ankle	Foot and ankle conditions	5 subscales / 42 items. Symptoms/stiffness (5+2), pain (9), ADL (17), function in sports and recreational activites (5), foot and ankle-related QoL (4).	0-4	Raw score of each subscale converted to 0 (worst) - 100 (best)
Foot and Ankle Ability Measure	Greve 2018, Turhan 2017	Physical function (disability)	Musculoskeletal disorders of the feet and ankle	2 subscales / ADL 21 items + sports 8 items	0-4	ADL 0-84 (worst), sports 0-32 (worst)
Global self-rated function	Büker 2017, Nilsson 2013	Ankle function	General	1 item	Very good, good, fair, poor, very poor	
Linear analgoue scale	Olerud-Molander 1984	Ankle function	General	1 item	15 cm long linear analogue scale	0-100 (best)
Lower Extremity Scale	Garratt 2018, Ponkilainen 2019, Repo 2018	Physical function (disability) in ADL	Musculoskeletal conditions or disorders in lower limb	20 items	0-4	0 (worst) - 80 (best)
Manchester Oxford Foot Questionnaire	McKeown 2021	Recovery in indivduals with foot and ankle conditions	Patients undergoing surgery on different regions of the foot and ankle	3 subscales / 16 items or summarized score (index). Walking/standing (7), pain (5), social interaction (4).	0-4	Converted to 0 (best) -100 (worst)

PROM	Reference	Construct	Target population	(Sub)scale(s) (number of items)	Response options	Range of scores/scoring
Olerud-Molander Ankle Score	Garratt 2018, Gausden 2018, Lin 2009	Physical disability	Follow-up of ankle fracture	1 scale / 9 items	Pain (0-25), stiffness (0-10), swelling (0-10), stair climbing (0-10), running (0-5), jumping (0-5), use of supports (0-10), work/activity (0-20)	0 (worst) - 100 (best)
Self-Reported Foot and Ankle Score	Garratt 2018	Pain, function, other	Osteoarthritis and inflammatory arthritis of the ankle and outcome of ankle surgery	12 items	0-4	12-60 (worst)
SF-12 Mental Component score	Shah 2007	Mental health	General	8 subscales / 12 items	Mix: binary, 1-3, 1-5, 1-6	Norm based, standardized mean score 50 SD 10. Range 0-100 (best)
SF-12 Physical Component score	Shah 2007, Turhan 2017	Physical health	General	8 subscales / 12 items	Mix: binary, 1-3, 1-5, 1-6	Norm based, standardized mean score 50 SD 10. Range 0-100 (best)
SF-36	Obremskey 2007, Ponzer 1999	Physical and mental health	General	8 subscales / 36 items	Mix: binary, 1-3, 1-5, 1-6	Each subscales: 0-100 (best)
SF-36 physical functioning	Garratt 2018, Suk 2013, Fang 2020	Physical function (disability)	General	10 items	1-3	0 -100 (best)

PROM	Reference	Construct	Target population	(Sub)scale(s) (number of items)	Response options	Range of scores/scoring
SF-36 Mental Component Score	Zelle 2017	Mental health	General	8 subscales / 35 items	Mix: binary, 1-3, 1-5, 1-6	Norm based, standardized mean score 50 SD 10. Range 0-100 (best)
SF-36 Physical Component Score	Zelle 2017	Physical health	General	8 subscales / 35 items	Mix: binary, 1-3, 1-5, 1-6	Norm based, standardized mean score 50 SD 10. Range 0-100 (best)
Visual Analogue Scale	Ponzer 1999	Mental/physical injury related health		1 item	100mm long line	0-100 (worst)
Visual Analogue Scale – Foot and Ankle	Ponkilainen 2019	Not defined	Foot and ankle patients	3 subscales (function, pain, other complaints) / 20 items	VAS 0-100mm	0 - 100 (best)

Abbreviations:
ADL = Activities of daily living
HrQoL = Health-related quality of life
SD = Standard deviation
SF = Short form
VAS = Visual Analogue Scale
