# Supplementary material for: Ankle fractures: a systematic review of patient-reported outcome measures and their measurement properties
Source: Qual Life Res. 2022 Jun 18;32(1):27–45. doi: 10.1007/s11136-022-03166-3 (PMC9829578; doi:10.1007/s11136-022-03166-3)
Supplement: Supplementary file 5 — Supplementary file5 (PDF 226 KB) [file 11136_2022_3166_MOESM5_ESM.pdf]

Article title

Ankle Fractures: A Systematic Review of Patient Reported Outcome Measures and their measurement properties

Journal name

Quality of Life Research

Author information

Michael Quan Nguyen[1,2], Ingvild Dalen[2,3], Marjolein Memelink Iversen[4,5], Knut Harboe[1,6], Aksel Paulsen[1,7]

[1]Department of Orthopedic Surgery, Stavanger University Hospital, Helse Stavanger HF.
[2]Department of Quality and Health Technology, Faculty of Health Sciences, University of Stavanger.
[3]Department of Research, Stavanger University Hospital, Helse Stavanger HF.
[4]Centre on Patient-reported Outcomes, Department of Research and Development, Haukeland University Hospital, Helse Bergen HF.
[5]Department of Health and Caring Sciences, Faculty of Health and Social Sciences, Western Norway University of Applied Sciences.
[6]Department of Clinical Medicine, Faculty of Medicine, University of Bergen.
[7]Department of Public Health, Faculty of Health Sciences, University of Stavanger.

Corresponding author:

Michael Quan Nguyen
E-mail: n.michael.quan@gmail.com
ORCID: 0000-0003-0270-9518

Online Resource 5 Summary of findings table

AMERICAN ACADEMY OF ORTHOPAEDIC SURGEONS FOOT AND ANKLE OUTCOMES QUESTIONNAIRE

Reference	Content validity		Structural validity		Internal consistency		Cross-cultural validity		Reliability		Measurement error		Criterion validity		H testing for construct validity		Responsiveness	
	MQ	R	MQ	R	MQ	R	MQ	R	MQ	R	MQ	R	MQ	R	MQ	R	MQ	R
Zelle 2017	D	+													A	H met: 4		
Summarized results	Only comprehensibility														H met: 4			
Overall rating	NA														Sufficient			
Quality of evidence	NA														Low; downgraded due to risk of bias and total sample size less than 100 (n = 83)			

ANKLE FRACTURE OUTCOME OF REHABILITATION

Reference	Content validity		Structural validity		Internal consistency		Cross-cultural validity		Reliability		Measurement error		Criterion validity		Hypothesis testing for construct validity		Responsiveness	
	MQ	R	MQ	R	MQ	R	MQ	R	MQ	R	MQ	R	MQ	R	MQ	R	MQ	R
McPhail 2014	I	+																
Summarized results	NA																	
Overall rating	Sufficient																	
Quality of evidence	Very low; PROM development study inadequate, only reviewers' ratings																	

FOOT AND ANKLE ABILITY MEASURE

Reference	Content validity		Structural validity		Internal consistency		Cross-cultural validity		Reliability		Measurement error		Criterion validity		H testing for construct validity		Responsiveness	
	MQ	R	MQ	R	MQ	R	MQ	R	MQ	R	MQ	R	MQ	R	MQ	R	MQ	R
Schultz 2020															I	NA		
Summarized results															NA			
Overall rating															?			
Quality of evidence															NA			

LOWER EXTREMITY FUNCTIONAL SCALE

Reference	Content validity		Structural validity		Internal consistency		Cross-cultural validity		Reliability		Measurement error		Criterion validity		H testing for construct validity		Responsiveness	
	MQ	R	MQ	R	MQ	R	MQ	R	MQ	R	MQ	R	MQ	R	MQ	R	MQ	R
Garratt 2018			V	(+) CFA: CFI 0.99/TLI 0.99/RMSEA 0.091	V	(+) Cα = 0.96			A	(+) ICC: 0.91	A	(?) SDC: 12.5			A	H met: 7 H unmet: 1		
Garratt 2018															D	H unmet: 1		
Lin 2009 (1)			V	(-)	V	(?)												
Lin 2009 (2)															V	H met: 2		
Lin 2009 (3)																	D	H met: 2 AUC: 0.79 and 0.84
Repo 2017	D	+	A	?	I	?			A	(+) ICC 0.93	A	?			A	H met: 4		
Repo 2019			A	-														
Summarized results	Only comprehensibility		+/-		?				ICC: 0.91-0.93		SDC: 12.5 MIC: unknown				H met: 13 H unmet: 2		H met: 2	
Overall rating	NA		Inconsistent		Indeterminate				Sufficient		Indeterminate				Sufficient 87% confirmed		Sufficient	
Quality of evidence	NA		NA		NA				High; Two studies of adequate quality		NA				High		Low	

THE MUNICH ANKLE QUESTIONNAIRE

Reference	Content validity		Structural validity		Internal consistency		Cross-cultural validity		Reliability		Measurement error		Criterion validity		H testing for construct validity		Responsiveness	
	MQ	R	MQ	R	MQ	R	MQ	R	MQ	R	MQ	R	MQ	R	MQ	R	MQ	R
Greve 2018					D	(?)			I	(+) ICC: 0.77-0.80					A	H met: 3	A	H met: 3; SCC pain, work/daily and movement/ROM: 0.42-0.47
Summarized results					?				ICC: 0.77-0.80						H met: 3		H met: 3	
Overall rating					Indeterminate				Sufficient						Sufficient		Sufficient	
Quality of evidence					NA				Very low						Moderate		Moderate	

OLERUD MOLANDER ANKLE SCORE

Reference	Content validity		Structural validity		Internal consistency		Cross-cultural validity		Reliability		Measurement error		Criterion validity		H testing for construct validity		Responsiveness	
	MQ	R	MQ	R	MQ	R	MQ	R	MQ	R	MQ	R	MQ	R	MQ	R	MQ	R
Büker 2017					V	(+) Cα = 0.76			I	(+) ICC: 0.942					A	H met: 6		
Lash 2002															I	NA		
Garratt 2018			V	(+) CFA: CFI 0.99/TLI 0.98/RMSEA 0.087	V	(+) Cα = 0.82			A	(+) ICC: 0.92	A	(-) SDC: 19.0 MIC: 9.7[a]			A	H met: 6 H unmet: 2		
Garratt 2018															D	H unmet: 1		
McKeown 2021			A	(?)	V	(+) Cα total = 0.76									A	H met: 8 H unmet: 4		
Nilsson 2013 (1)					V	(+) Cα = 0.76			D	(+) ICC: 0.94	D	(-) SDC: 12.0 MIC: 9.7[a]						
Nilsson 2013 (2)															I	NA		
Nilsson 2013 (3)															A	H met: 5		
Olerud-Molander 1984															I	NA		
Ponzer 1999															D	H met: 8 H unmet: 2		
Shah 2007															A	H met: 1 H unmet: 1		
Turhan 2017	D	+			V	(+) Cα =0.84			I	(+) ICC: 0.98	I	(+) SDC: 9.1 MIC: 9.7[a]			A	H met: 2 H unmet: 2		
Summarized results	Only comprehensibility		Unidimensional		Cα: 0.76-0.84				ICC: 0.92-0.98		SDC: 9.1-19.0 MIC 9.7[a]				H met: 36 H unmet: 12			
Overall rating	NA		Sufficient		Sufficient				Sufficient		Insufficient				Sufficient 75% confirmed			
Quality of evidence	NA		High; one very good study		High; multiple very good studies; consistent results				Moderate; only one study of adequate quality		Very low; only one study of adequate quality, one MIC, and indirectness (follow-up time 4.3 years vs. 16 weeks)				High; multiple studies of adequate quality			

PROMIS LE CAT

Reference	Content validity		Structural validity		Internal consistency		Cross-cultural validity		Reliability		Measurement error		Criterion validity		H testing for construct validity		Responsiveness	
	MQ	R	MQ	R	MQ	R	MQ	R	MQ	R	MQ	R	MQ	R	MQ	R	MQ	R
Gausden 2018															D	H met: 5<br>H unmet: 1		
Summarized results															H met: 5<br>H unmet: 1			
Overall rating															Sufficient			
Quality of evidence															Low			

PROMIS PF VER 1.2 CAT

Reference	Content validity		Structural validity		Internal consistency		Cross-cultural validity		Reliability		Measurement error		Criterion validity		H testing for construct validity		Responsiveness	
	MQ	R	MQ	R	MQ	R	MQ	R	MQ	R	MQ	R	MQ	R	MQ	R	MQ	R
Gausden 2018															D	H met: 4<br>H unmet: 2		
Summarized results															H met: 4<br>H unmet: 2			
Overall rating															Insufficient			
Quality of evidence															Low			

SELF-REPORTED FOOT AND ANKLE SCORE

Reference	Content validity		Structural validity		Internal consistency		Cross-cultural validity		Reliability		Measurement error		Criterion validity		H testing for construct validity		Responsiveness	
	MQ	R	MQ	R	MQ	R	MQ	R	MQ	R	MQ	R	MQ	R	MQ	R	MQ	R
Erichsen 2021 (1)	I	+																
Erichsen 2021 (2)					V	(+) Cα = 0.93									D	H unmet: 1		
Erichsen 2021 (3)									I	(+) ICC: 0.93	I	(-) SDC: 6.8 MIC: 5						
Garratt 2018			V	(+) CFA: CFI 0.99/TLI 0.99/RMSEA 0.063	V	(+) Cα = 0.93			A	(+) ICC: 0.93	A	(-) SDC: 6.6 MIC: 5[b]			A	H met: 6 H unmet: 2		
Summarized results	Only comprehensibility rated		Unidimensional		Cα = 0.93				ICC: 0.93		SDC: 6.6-6.8 MIC: 5				H met: 6 H unmet: 3			
Overall rating	NA		Sufficient		Sufficient				Sufficient		Insufficient				Sufficient 75% confirmed (based on at least adequate MQ: H met: 6, H unmet: 2)			
Quality of evidence	NA		High		High				Moderate; only one study of adequate quality		Very low; MIC based on only one study and not adequately performed				Moderate			

SHORT MUSCULOSKELETAL FUNCTIONAL ASSESSMENT

Reference	Content validity		Structural validity		Internal consistency		Cross-cultural validity		Reliability		Measurement error		Criterion validity		H testing for construct validity		Responsiveness	
	MQ	R	MQ	R	MQ	R	MQ	R	MQ	R	MQ	R	MQ	R	MQ	R	MQ	R
Obremskey 2007															I	H met: 8		
Summarized results															H met: 8			
Overall rating															Sufficient			
Quality of evidence															Very low			

TRAUMA EXPECTATION FACTOR OUTCOMES MEASURE

Reference	Content validity		Structural validity		Internal consistency		Cross-cultural validity		Reliability		Measurement error		Criterion validity		H testing for construct validity		Responsiveness	
	MQ	R	MQ	R	MQ	R	MQ	R	MQ	R	MQ	R	MQ	R	MQ	R	MQ	R
Suk 2013	I	+/-			I	?			I	(+) ICC: 0.92-0.96					I	H met: 22<br>H unmet: 2	I	?
Fang 2020					I	?			I	(+) ICC: 0.93-0.94					I	H met: 3<br>H unmet: 13	I	?
Summarized results					?				ICC: 0.92-0.96						H met: 25<br>H unmet: 15		?	
Overall rating	Inconsistent				Indeterminate				Sufficient						Insufficient		Indeterminate	
Quality of evidence	Very low: PROM development study inadequate. only reviewer's ratings				NA				Low						Low		NA	

VISUAL ANALOGUE SCALE FOOT AND ANKLE

Reference	Content validity		Structural validity		Internal consistency		Cross-cultural validity		Reliability		Measurement error		Criterion validity		H testing for construct validity		Responsiveness	
	MQ	R	MQ	R	MQ	R	MQ	R	MQ	R	MQ	R	MQ	R	MQ	R	MQ	R
Repo 2018	D	+	D	?	I	?			A	(+) ICC: 0.97					I	H met: 2		
Summarized results	Only comprehensibility		?		?				ICC: 0.97						H met: 2			
Overall rating	NA		Indeterminate		Indeterminate				Sufficient						Sufficient			
Quality of evidence	NA		NA		NA				Moderate						Very low			

WESTERN ONTARIO AND MCMASTER UNIVERSITIES OSTEOARTHRITIS INDEX VERSION 3.0 FOOT/ANKLE

Reference	Content validity		Structural validity		Internal consistency		Cross-cultural validity		Reliability		Measurement error		Criterion validity		H testing for construct validity		Responsiveness	
	MQ	R	MQ	R	MQ	R	MQ	R	MQ	R	MQ	R	MQ	R	MQ	R	MQ	R
Ponkilainen 2019 (1)					D	(+)<br>Cα-pain = 0.95<br>Cα-stiffness = 0.86<br>Cα-physical function = 0.98									A	H met: 1		
Ponkilainen 2019 (2)															V	H met: 2		
Ponkilainen 2019 (3)															I	H met: 1		
Summarized results					NA										H met: 4			
Overall rating					NA; lack of evidence on structural validity										Sufficient			
Quality of evidence					NA										High			

[a] MIC from study of McKeown et al.
[b] MIC from study of Erichsen et al.

Abbreviations:
H = Hypothesis
MQ = methodological quality
R = rating
V = very good
A = adequate
D = doubtful
I = inadequate
? = indeterminate
+ = sufficient
- = insufficient
+/- = inconsistent
NA = not applicable
CFI = comparative fit index
TLI = Tucker-Lewis index
RMSEA = Root Mean Square Error of Approximation
Cα = Cronbach's Alpha
ICC = intraclass correlation coefficient
SDC = smallest detectable change
MIC = minimal important change
PROMIS = Patient-Reported Outcomes Measurement Information System
CAT = Computer Adaptive Test
PF = Physical Function
LE = Lower Extremity
AUC = area under curve
