# Supplementary material for: Ankle fractures: a systematic review of patient-reported outcome measures and their measurement properties
Source: Qual Life Res. 2022 Jun 18;32(1):27–45. doi: 10.1007/s11136-022-03166-3 (PMC9829578; doi:10.1007/s11136-022-03166-3)
Supplement: Supplementary file 6 — Supplementary file6 (PDF 95 KB) [file 11136_2022_3166_MOESM6_ESM.pdf]

**Article title**

# Ankle Fractures: A Systematic Review of Patient Reported Outcome Measures and their measurement properties

**Journal name**

Quality of Life Research

**Author information**

Michael Quan Nguyen[1,2], Ingvild Dalen[2,3], Marjolein Memelink Iversen[4,5], Knut Harboe[1,6], Aksel Paulsen[1,7]

[1]Department of Orthopedic Surgery, Stavanger University Hospital, Helse Stavanger HF.
[2]Department of Quality and Health Technology, Faculty of Health Sciences, University of Stavanger.
[3]Department of Research, Stavanger University Hospital, Helse Stavanger HF.
[4]Centre on Patient-reported Outcomes, Department of Research and Development,
Haukeland University Hospital, Helse Bergen HF.
[5]Department of Health and Caring Sciences, Faculty of Health and Social Sciences, Western Norway University of Applied Sciences.
[6]Department of Clinical Medicine, Faculty of Medicine, University of Bergen.
[7]Department of Public Health, Faculty of Health Sciences, University of Stavanger.

**Corresponding author:**

Michael Quan Nguyen
E-mail: n.michael.quan@gmail.com
ORCID: 0000-0003-0270-9518

**Online Resource 6** Table on interpretability

| Reference | PROM | Distribution of scores in the population | Missing (%) | Floor and ceiling effects | MIC |
|---|---|---|---|---|---|
| Büker 2017 | OMAS | Mean 73 ± 23 (range 10-100) | ? | ? | ? |
| Erichsen 2021 (2) | SEFAS | Mean 28 ± 9.6 | ? | 6 weeks: 22.4% (floor) | Mean 5 ±1.5 |
| Fang 2020 | TEFTOM | TOM 12 months mean 34 ± 5.7 | ? | ? | ? |
| Garratt 2018 | LEFS | Mean 67 ± 15 | 4.2 | ? | ? |
| Garratt 2018 | OMAS | Mean 74 ± 25 | 17.3 | ? | ? |
| Garratt 2018 | SEFAS | Mean 81 ± 19 | 8.5 | ? | ? |
| Gausden 2018 | PROMIS LE CAT | ? | ? | No | 3.7 (distribution-based) |
| Gausden 2018 | PROMIS PF ver 1.2 CAT | ? | ? | No | 4.3 (distribution-based) |
| Greve 2018 | MAQ | Mean 76 ± 19 | ? | No | |
| Lash 2002 | OMAS | Mean 71 ± 22 | ? | ? | ? |
| Lin 2009 (1) | LEFS | Baseline: mean 31 ± 12; 4-6 w: mean 54 ± 13; 24-26 w: mean 69 ± 12 | 5.8 | No | ? |
| Lin 2009 (2) | LEFS | 6 w: mean 51 ± 13; 26 w: mean 66 ± 12 | ? | ? | ? |
| McKeown 2021 | OMAS | ? | ? | No | Mean 9.7 ± 15 |
| McPhail 2014 | A-FORM v1.0 | ? | ? | ? | ? |
| Nilsson 2013 (1) | OMAS | First assessment mean 75 ± 19; second assessment mean 77 ± 18 | ? | No | ? |
| Nilsson 2013 (2) | OMAS | ? | ? | ? | ? |
| Nilsson 2013 (3) | OMAS | Median 80 (IQR 25, range 30-100) | ? | No | ? |
| Obremskey 2007 | SMFA | Dysfunction index mean 17 ± 15; Bother index mean 18 ± 18 | < 5 | No | ? |
| Olerud-Molander 1984 | OMAS | ? | ? | ? | ? |

| Reference | PROM | Distribution of scores in the population | Missing (%) | Floor and ceiling effects | MIC |
|---|---|---|---|---|---|
| Ponkilainen 2019 | WOMAC version 3.0 foot/ankle | Index median 8 (IQR 22); Pain median 6 (IQR 16); Stiffness median 8 (IQR 33); PF median 6 (IQR 13) | ? | No | ? |
| Ponzer 1999 | OMAS | Mean 84 ± 23 (range 10-100) | ? | ? | ? |
| Repo 2017 | LEFS | Mean 66 ± 15 | ? | 17% (ceiling) | ? |
| Repo 2018 | VAS-FA | Pain mean 73 ± 27; Function mean 76 ± 24; Other complaints mean 74 ± 23 | ? | No | ? |
| Repo 2019 | LEFS | ? | ? | ? | ? |
| Schultz 2020 | FAAM | ? | ? | ? | ? |
| Shah 2007 | OMAS | Weber B mean 75 (95% CI 70-82); Weber C mean 73 (95% CI 61-86) | ? | ? | ? |
| Suk 2013 | TEFTOM | TOM 12 months: mean 33 (95% CI: 32-34) | ? | No | ? |
| Turhan 2017 | OMAS | Mean 74 ± 24 | ? | First assessment: 27% (ceiling); Second assessment: 29% (ceiling) | ? |
| Zelle 2017 | AAOS-FAOQ | Mean 48 ± 24 | ? | ? | ? |

? = Not reported

Abbreviations:
AAOS-FAOQ = American Academy of Orthopaedic Surgeons Foot and Ankle Outcomes Questionnaire
A-FORM = Ankle Fracture Outcome of Rehabilitation Measure
CAT = Computer Adaptive Test
FAAM = Foot and Ankle Ability Measure
IQR = Interquartile Range
LE = Lower Extremity
LEFS = Lower Extremity Functional Scale
MAQ = Munich Ankle Questionnaire
MIC = Minimal Important Change
OMAS = Olerud-Molander Ankle Score
PF = Physical Function
PROMIS = Patient-Reported Outcomes Measurement Information System
SEFAS = Self-Reported Foot and Ankle Score
SMFA = Short Musculoskeletal Function Assessment
TEFTOM = Trauma Expectation Factor Trauma Outcome Measure
VAS-FA = Visual Analogue Scale Foot and Ankle
WOMAC = Western Ontario and McMaster Universities Osteoarthritis Index
